# eIF4E and Ezrin cooperate in pseudopods to drive a localized migratory translation program in acute myeloid leukemia

**DOI:** 10.64898/2026.02.21.707190

**Published:** 2026-02-23

**Authors:** Biljana Culjkovic-Kraljacic, Leandro Marcelo Martinez, Alyssa Retiz, Samuel Perron, Naiyu Shi, Caleb Embree, Winnie Yip, Vicenta Trujillo-Alonso, Emma Lassman, Marysol Chu Carty, Kata Alilovic, Sebastien Carreno, Gail J Roboz, Monica L. Guzman, Katherine L.B. Borden

**Affiliations:** 1 Department of Pathology and Cell Biology & Institute for Research in Immunology and Cancer, Université de Montréal, Québec, Canada.; 2 Department of Pharmacology and Robert H. Lurie Cancer Centre, Northwestern University, Chicago, Illinois, USA; 3 Division of Hematology/Medical Oncology, and Department of Medicine, Sandra & Edward Meyer Cancer Center, Weill Cornell Medicine, New York, NY, USA.; 4 Pharmacology Department, Weill Cornell Medicine, New York, NY, USA

## Abstract

Aggressive subtypes of acute myeloid leukemia (AML) are characterized by increased migratory behavior and poor prognosis prioritizing the need for uncovering relevant mechanisms. While attributed to transcriptional changes, these AMLs manifest dysregulated eIF4E implicating disrupted mRNA metabolism. Here, we observed in AML mouse models, patient specimens, and cell lines that eIF4E drives motility, colonization, engraftment and AML progression. AML cells migrate utilizing Ezrin-positive pseudopods. Unexpectedly, we discovered that eIF4E interacts with Ezrin, that these physically associated factors are required and cooperated to drive an on-demand translation program in pseudopods for motility. Indeed, pseudopods were sites of eIF4E- and Ezrin-dependent translation by implementing the first method to directly mark active ribosomes *in situ* (Visualizing Translation Activity using RiboLace, VISTA-R). Biochemically, Ezrin bound eIF4E, ribosomal components, and mRNAs consistent with our observed Ezrin-dependent modulation of protein production. This unprecedented physical coupling of motility and translation provisions migratory sites to sustain AML progression.

## Graphical Abstract

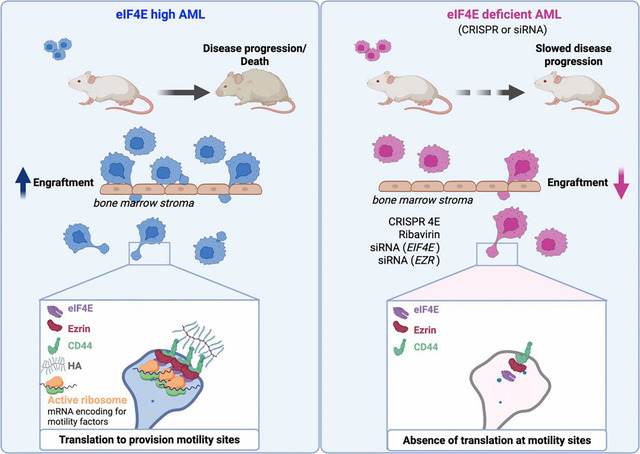

## Introduction

Acute myeloid leukemia (AML) is a malignancy characterized by the accumulation of immature myeloid cells, disrupting normal hematopoiesis and ultimately leads to bone marrow (BM) failure and death. Despite advancements in treatment, survival rates for AML remain poor, particularly among older adults^1^. A significant factor in leukemia initiation and persistence is the interaction between AML cells and the BM niche^2,3,4^. This process involves the ability of AML cells to home, infiltrate, and engraft in the BM^5^. This process in reverse provides the pathway for extravasation with implications for extramodular leukemia observed in ~25–50% of AML patients^6,7^. In solid tumors, dysregulation of the eukaryotic translation initiation factor eIF4E is strongly correlated with increased metastases in both patients and mouse models^8^. Moreover, eIF4E is linked to poor patient outcomes across various cancers, including AML where it is also linked to highly migratory AML subtypes^9–14^. eIF4E also plays a causative role in HoxA9 mediated AML^15–17^. Despite this clear clinical correlation, the mechanisms by which eIF4E promotes infiltration of malignant cells in solid or hematological malignancies have not been well understood.

As alluded to above, a subset of AML patients are characterized by substantially elevated eIF4E levels and in many cases their cells have abnormally large nuclear bodies relative to CD34+ BM cells from healthy donors^12,13,18,19^ or to AML patients with normal eIF4E levels^12,13,18,19^. High-eIF4E AML is found in patients with French American British (FAB) M4/M5 AML subtypes which are associated with increased infiltration as well as in some patients with M1/M2 AML subtypes^12,13,18,19^. While FAB subtypes are no longer used to classify AML, these data showcase that a substantial fraction of AML patients have elevated eIF4E. Nuclear enrichment of eIF4E in these specimens correlated with elevated eIF4E-dependent mRNA export relative to CD34+ cells from healthy donors or normal-eIF4E AML^12,13,18,19^. Survival analysis of a 609 AML patient cohort (Leucegene.ca) indicated that high-eIF4E AML patients had substantially reduced survival relative to others (p=.0438)^9^. Thus, eIF4E alone is a significant indicator of poor prognosis in AML. Notably, eIF4E associates with target mRNAs via their methyl-7-guanosine (m^7^G) cap on the 5’ ends of transcripts in both the nuclear and cytoplasmic compartments^8^. The antiviral drug ribavirin acts as a m^7^G-cap competitor by directly binding eIF4E, as shown by NMR and other biophysical techniques^20–22^. Over 30 groups have demonstrated ribavirin inhibits eIF4E’s activities in mRNA export, translation, and oncogenic transformation e.g.,^19–21,23–29^. This prompted three clinical trials in AML using ribavirin to treat refractory/relapsed high-eIF4E AML patients leading to objective clinical responses including complete remissions correlating with molecular targeting of eIF4E^12–14^. Early-stage clinical trials targeting eIF4E with ribavirin in combination with other drugs have also been carried out in castration-resistant prostate cancer and oral cancers where eIF4E targeting was demonstrated in a subset of patients corresponding to objective clinical benefit^30–32^. Relapse resulted from chemical deactivation of ribavirin or impaired drug retention and uptake^12–14,20,33–35^.

AML cells employ an ameboid-like strategy reliant on pseudopod-like protrusions for movement through BM stroma whereby the surfaces of these structures are decorated with factors involved in stromal interactions^36,37,38^. These factors include the CD44 and glycosaminoglycan hyaluronan (HA) which are essential for the homing, migration and infiltration of AML cells to the BM niche^39,40^. Communication between AML cells and the BM matrix is critical for initiating programs to promote migration, BM infiltration, and disease progression^36^. CD44 and the HA play critical roles in these facets of motility in AML *e.g.*, CD44 on the surface of AML cells enables embedding into the HA-rich BM niche; indeed, elevated HA levels correlate with increased adhesion of hematopoietic cells to the niche^40^. The HA-CD44 interaction stimulates signaling events which promote migratory behavior of AML cells and increase their production of proteases which in turn degrade cell adhesion junctions thereby generating space through which cells migrate^40^. HA is comprised of repeating disaccharide units of UDP-glucuronic acid and UDP-N-acetyl glucosamine forming chains up to 10^7^ Da in size^41^. HA is generally extruded into the pericellular space, forming a major component of the BM stroma but is also found encapsulating high-eIF4E AML patient cells suggestive of autocrine signaling to CD44^41,42^. Aside from the protease release noted above, HA binding to CD44 promotes intracellular signaling which drives the mechanics underpinning morphological rearrangements required for cells to invade. CD44 conveys these signals through its direct interaction with Ezrin, a member of the ERM family (Ezrin, Radixin, Moesin) or in some instances via the other ERM proteins^43,44^. Ezrin directly links CD44 to cortical Actin, i.e. Actin associated with the plasma membrane, which provides the mechanics for the physical deformations of the membrane required for pseudopod formation and motility of AML and other cancer cells^43,44^.

Previous studies provided a link between eIF4E, motility in solid cancer cell lines and its capacity to drive the production of CD44 and HA^34^. This capacity had been attributed to the nuclear fraction of eIF4E which has multiple functions including enhanced mRNA export described above. Indeed, eIF4E drives the mRNA export, and hence cytoplasmic availability, of all the enzymes involved in CD44 and HA production in high-eIF4E AML and osteosarcoma cells^45^. Additionally, a few of these transcripts including CD44 also have their translation efficiency (number of ribosomes per transcript) increased by eIF4E as well as undergo eIF4E-dependent splicing^9,45^. Genetic or pharmacological targeting of CD44 or independently HA was sufficient to reduce eIF4E-dependent motility without impacting cell viability in osteosarcoma or breast cancer cells^34,45^. While eIF4E’s capacity to control the production of motility factors at multiple levels is undoubtedly important, its impact on motility in physiological settings was unknown nor was there an understanding of whether there was direct interplay between eIF4E and the motility machinery.

Here, we identified eIF4E-dependent post-transcriptional control as an important driver of AML motility *in vivo* and dissect the underlying molecular mechanisms. Indeed, we find that eIF4E controls migratory features of AML cells *in vitro* and *in vivo*. Moreover, we show that this eIF4E activity required the motility factor Ezrin which we surprisingly found physically associated with eIF4E, mRNAs encoding the Ezrin-CD44-HA circuit, and ribosomal components indicative of a previously unknown role for the motility machinery in translation. The physical association of Ezrin with the translation machinery coupled to its enrichment in pseudopods in motile AML cells led us to identify a new form of localized oncogenic translation. We showed that Ezrin-positive pseudopods also contained eIF4E and additionally, active ribosomes as directly detected for the first time using our method, VISTA-R (*Vis*ualizing *T*ranslation *A*ctivity using *R*iboLace). We refer to these structures as translationally active pseudopods (T-PODs) and their translation activity depended on both Ezrin and eIF4E. We investigated the role of T-PODs as new sites for on-demand translation of motility factors at sites of oncogenic migration. Our findings indicate that the translation machinery is positioned to provide motility factors to the pseudopods revealing a novel strategy to supply sites of oncogenic migration: pseudopod localized active translation.

## Results

### eIF4E drives AML cell migration, infiltration and disease progression

**Figure 1.**
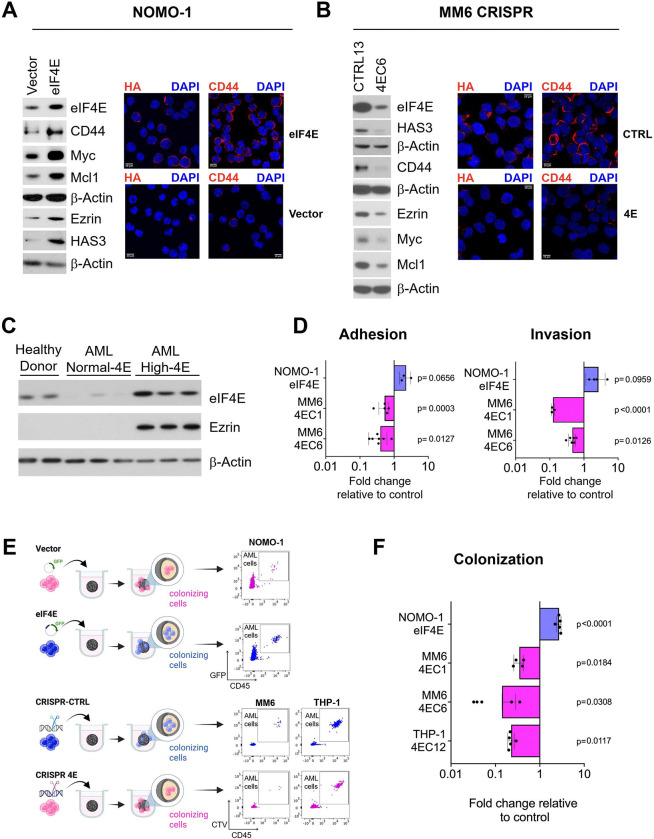
eIF4E controls cell adhesion, migration and colonization *in vitro*. **A, B.** The impact on the Ezrin-CD44-HA axis of NOMO-1 eIF4E overexpression (eIF4E) compared to vector control (A) or of MM6 CRISPR-4E cells relative to MM6 CRISPR-CTRL cells (B). **Left panels.** Western blots of eIF4E and Ezrin-CD44-HA axis. Corresponding b-Actin shown for loading controls. **Right panels**. Confocal micrographs (single section through the plane of cells) of the indicated cells stained for HA or CD44 shown in red. DAPI shown in blue. Scale bar = 10μm. **C.** Western blot of primary specimens from AML patients (with normal or high-eIF4E) and CD34^+^ from healthy donors showing expression of eIF4E and Ezrin. b-Actin is provided as a loading control. Each lane represents a different individual. **D.** Adhesion (left) and invasion (right) capacity using HS-5 stromal cells of NOMO-1 and MM6 cell lines as a function of genetic eIF4E overexpression or CRISPR knockdown respectively. Data is presented as fold change relative to their corresponding controls. Each symbol represents an independent experiment performed with replicates. The bar represents the mean with standard deviations and p-values calculated with two tailed paired T test. **E.** Schematic representation of colonization assay into mesenchymal stromal cell spheroid model mimicking the bone marrow niche. **F.** Quantification of AML cell colonization capacity relative to their corresponding controls. Each symbol represents an experimental replicate. The bar represents the mean with standard deviations and p-values, Welch’s t test.

The mechanisms that control the ability of AML cells to migrate and infiltrate are not well understood, specifically the extent to which these depend upon post-transcriptional control. To address this question, we examined whether eIF4E activity impacted AML cell motility and infiltration. For this purpose, we generated two AML cell line models. For gain of function, we employed NOMO-1 AML cells since these have eIF4E levels similar to healthy donor CD34+ cells^46^ (Supplemental Figure 1A) allowing us to assess the effects of stable eIF4E overexpression pools (NOMO-1 eIF4E) relative to vector controls (NOMO-1 Vector). Here, eIF4E-overepression was sufficient to recapitulate the elevated, substantially nuclear eIF4E phenotypes typical of high-eIF4E AML primary specimens^46^. eIF4E was elevated more than 2-fold in NOMO-1 eIF4E cells versus NOMO-1 Vector ^46^ which is well within the range of its elevation up to 8-fold observed in AML patients to date^12,13^ (Figure 1A, Supplemental Figure 1C). For loss of function, we used CRISPR to deplete eIF4E in Mono-Mac-6 (MM6) AML cells generating clonal MM6 CRISPR 4E cell lines. Parental MM6 cells have highly elevated eIF4E levels with substantial nuclear localization (Supplemental Figure1A and B) similar to those in high-eIF4E AML patients. Reduction of eIF4E in MM6 CRISPR 4E cell lines was ~3 fold assessing multiple clones (Figure1B, Supplemental Figure 1D) and knockdown was confirmed to be heterozygous by DNA sequencing. Notably, complete deletion of eIF4E is lethal. CRISPR control (CRISPR-CTRL) clonal cell lines were generated *via* sgRNAs to Azami-green fluorescent protein from coral as we did previously^47^.

Given the role of the Ezrin-CD44-HA axis in motility, we assessed whether their production was eIF4E-dependent in AML. We observed that elevation of eIF4E in NOMO-1 eIF4E cells relative to NOMO-1 Vector led to increased HAS3, CD44 and Ezrin protein levels as well as positive controls (Myc and Mcl1) but did not impact b-Actin, which is known not to be an eIF4E target (Figure 1A and Supplemental Figure 1C)^45^. Conversely, MM6 CRISPR 4E cell lines had substantially reduced levels of HAS3, CD44 and Ezrin versus CRISPR-CTRL using multiple clones giving highly similar results (Figure 1B; Supplemental Figure 1D and 1G). Next, we set out to monitor HA carbohydrate levels which we reasoned would be sensitive to eIF4E given the impact of eIF4E on several of the HA biosynthetic enzymes including HAS3, using fluorescently labelled HA-binding protein and confocal microscopy^45^. Here, HA levels were substantially elevated in NOMO-1 eIF4E cells versus NOMO-1 Vector; and conversely, HA levels were reduced in MM6 CRISPR 4E versus CRISPR-CTRL cell lines (Figure 1A–B). HA coated the cells consistent with our previous studies that HA was both elevated and coated leukemic cells in primary high-eIF4E AML patient specimens relative to CD34+ cells from healthy donors as well as in parental MM6 cells^45^. Importantly, treatment with hyaluronidase (HAse) eliminated the HA signal indicating that the staining was specific (Supplemental Figure 1E). Similar to genetic targeting, in parental MM6 cells, Ezrin and HAS3 protein levels were reduced by ribavirin as were positive controls Myc and Mcl1 while, the negative control, b-Actin was unaffected (Supplemental Figure 1F). In high-eIF4E AML cells, CD44 is elevated and forms surface coats (Figure 1A–B) consistent with its role in HA-mediated extracellular signaling^45^. Finally, we observed that Ezrin is highly elevated in primary high-eIF4E AML specimens relative to normal-eIF4E AML patients specimens or CD34+ cells from healthy donors (Figure 1C), consistent with these having elevated eIF4E and HA^45^; b-Actin is shown as a loading control. Thus, consistent with nuclear eIF4E RNA immunoprecipitation (RIP) and mRNA export data^45^, eIF4E drives protein production of Ezrin as well as of HAS3, CD44 in AML cells.

Since eIF4E modulates production of the Ezrin-CD44-HA axis in AML cells (Figure 1A–B), we investigated whether eIF4E played a role in AML motility by performing *in vitro* adhesion and invasion assays. For these assays, we employed the HS-5 human cell line as it recapitulates the major features of primary human BM mesenchymal stromal cells^48^. Adhesion assays were carried out by allowing AML cells pre-stained with fluorescent dye [DILC12(3)] to incubate with a monolayer of HS-5 cells for 1 hour, wash and then observe the number of cells retained on the stroma^45^. For invasion, pre-stained AML cells were loaded into the upper compartment of a Boyden chamber with HS-5 coated inserts and C-C motif chemokine ligand 2 (CCL2, also known as monocyte attractant protein 1, MCP-1) in the lower chamber as a chemoattractant for 24 hours. These AML cell lines double every ~36–60 hours and thus proliferative effects do not contribute to these results. Experiments with pools of stable NOMO-1 eIF4E and NOMO-1 Vector cells were conducted in biological replicates and different clones for MM6 CRISPR (4E and CTRL) cell lines were used. We observed that NOMO-1 eIF4E cells had ~2-fold increased adhesion relative to NOMO-1 Vector, and ~2-fold increased invasion through HS-5 stroma (Figure 1D). Conversely, MM6 CRISPR 4E cells had ~2.3-fold reduced adhesion and ~2.3-fold decreased invasion versus CRISPR-CTRL cells (Figure 1D). Plating controls were used to ensure equal loading of cells in both adhesion and invasion experiments (see Materials and Methods). In liquid culture, there were undetectable differences in proliferation for NOMO-1 eIF4E versus NOMO-1 Vector or MM6 CRISPR 4E over MM6 CRISPR-CTRL indicating that observed changes in motility are not due to differences in proliferation or survival (data not shown). In all, these studies reveal a major role for eIF4E in AML cell migration *in vitro*.

To build on these findings, we explored the eIF4E-dependency of AML infiltration in a system that mimics specialized niche structures and cell-to-cell intercommunication known to drive malignant hematopoiesis^49^. For this purpose, we used an *in vitro* functional 3D hematopoietic-mesenchymal co-culture system (spheroid) consisting of HS-5 and AML cells. Leukemia cells with infiltrating/colonizing properties are scored based on their localization inside the 3D structure (Figure 1E). Thus, using this system, we examined two different clones of MM6 CRISPR 4E cells which had between 3- and 7-fold reduced capacity for colonization on day 1 compared to two MM6 CRISPR-CTRL clonal cell lines (Figure 1F). We extended this into another high-eIF4E AML cell line THP-1 which were previously characterized by a >3-fold level of intrinsic eIF4E elevation relative to healthy donor specimens^19^. We generated THP-1 CRISPR 4E (THP-1 4EC12) and CRISPR-CTRL cells (THP-1 CTRL10) as for MM6 cells. Western blots confirmed knockdown of eIF4E corresponded to reduction in CD44, Ezrin and Myc in THP-1 CRISPR 4E cells (Figure 1H). This data is consistent with observations in CRISPR 4E THP-1 cells where we observed a 5-fold decrease in colonization relative to THP-1 CRISPR-CTRL cells and consistently, we found that NOMO-1 eIF4E cells had a 2.9-fold increase in colonization capacity relative to Vector controls. Taken together, we demonstrated that eIF4E modulation impacts the ability of leukemia cells to invade and colonize the spheroid which represents the BM niche.

**Figure 2.**
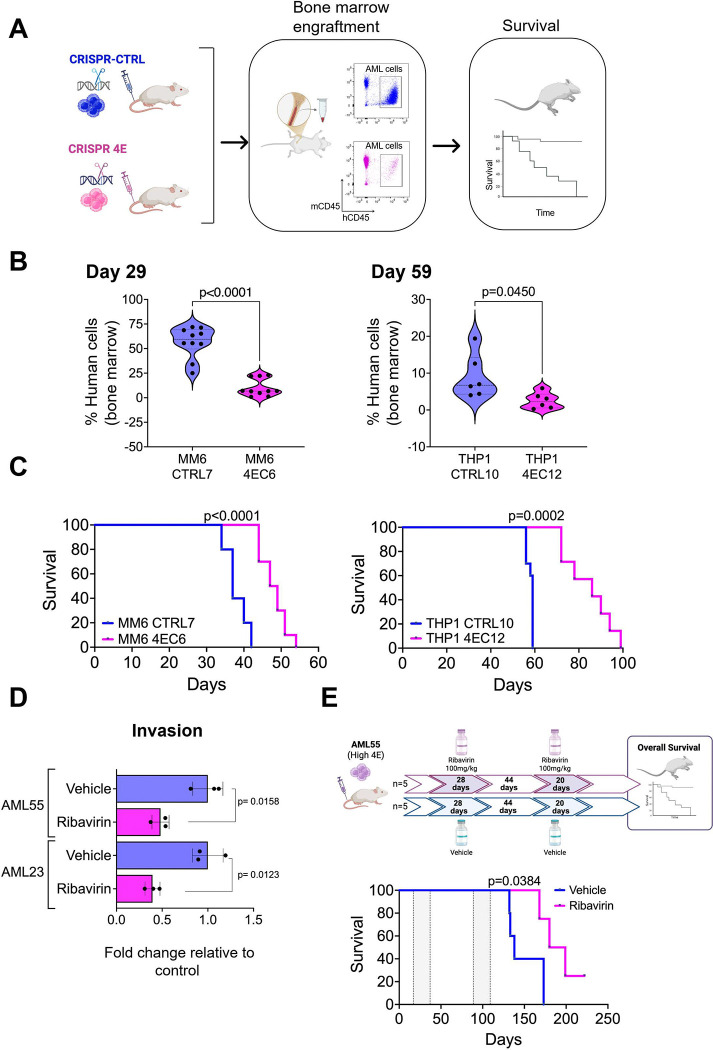
Depletion of eIF4E reduces engraftment and improves overall survival in AML cell line-derived xenograft (CDX) mouse models. **A**. Schematic representation of experimental design using CDX mouse models. **B.** Percent human cells in the bone marrow at the indicated timepoint assessing the AML engraftment capacity for the indicated cell lines, each dot represents an animal. Violin plot representing the median and quartiles, p values calculated with unpaired two tailed t test and Welch’s t test. **C.** Kaplan–Meier curves comparing overall survival of mice transplanted with the indicated cell lines. Log-rank (Mantel-Cox) test applied. **D.** invasion capacity through HS-5 stromal cells of the indicated primary AML samples after exposure to ribavirin or vehicle control. Data is presented as fold change relative to vehicle control. Each symbol is an independent experiment performed with replicates. The bar represents the mean with standard deviations and p-values calculated with Welch’s t test (two tailed). **E.** Pharmacological targeting of eIF4E with Ribavirin reduces leukemia burden and improves overall survival in an AML patient-derived xenograft (PDX) mouse model. Diagram illustrating the treatment regimen (top panel). Kaplan–Meier curves comparing overall survival of PDX mice with the indicated treatments. Log-rank (Mantel-Cox) test applied (bottom).

As we previously showed that colonizing cells in this model reflect cells with increased engraftment capacity in xenografts^49^, we monitored the impact of eIF4E depletion on the engraftment of AML cells to the BM niche employing the two MM6 and one THP-1 clonal cell lines for CRISPR 4E and CRISPR-CTRL using xenotransplantation into immunodeficient mice. We evaluated AML cell engraftment in BM at a timepoint when the control animals were expected to succumb to the disease (Figure 2A and Supplemental Figure 2A and 3A). We found a significant decrease in engraftment in mice transplanted with MM6 CRISPR 4E (clone 4EC6) cells compared to MM6 CRISPR-CTRL (clone CTRL7) cells [% of human cells in BM (mean ± SE): 10.13 ± 2.78 (n=10) vs. 56.53 ± 4.98 (n=10), respectively, p<0.0001] (Figure 2B). These results were corroborated on a different set of clonal cell lines, MM6 CRISPR 4E (clone 4EC1) cells compared to MM6 CRISPR-CTRL (clone CTRL13) cells [% of human cells in BM (mean ± SE): 0.26 ± 0.14 (n=5) vs. 5.20 ± 1.51 (n=5), respectively, p=0.0079] Supplemental Figure 2B. Importantly, these observations were validated using another set of CRISPR 4E and CRISPR-CTRL in THP-1 cells. THP-1 cells needed longer time to engraft but still caused disease in the animals. Thus, when we first evaluated engraftment at day 30 prior to any symptoms of disease, we found that 80% of the animals transplanted with THP-1 CRISPR-CTRL (clone CTRL10) had detectable human cells in the BM, in stark contrast with 10% of the THP-1 CRISPR 4E (clone 4EC12, Supplemental Figure 3B). Next, we waited until the control animals started to display signs of disease, at this point day 56. As we sacrificed 3 succumbing THP-1 CRISPR-CTRL mice, we took 3 CRISPR 4E mice to compare the engraftment levels in BM, and spleen, confirming a higher engraftment of human cells in the CTRL mice when compared with CRISPR 4E (Supplemental Figure 3C). At day 59, all CTRL animals had to be sacrificed due to disease progression, we isolated the BM from these animals to compare with BM aspirates from all CRISPR 4E surviving mice (with no signs of disease). We again observed a significant decrease in engraftment in mice transplanted with THP-1 CRISPR 4E (clone 4EC12) cells compared to THP-1 CRISPR-CTRL (clone CTRL10) cells: [% of human cells in BM (mean ± SE): 2.5 ± 0.87 (n=6) vs. 8.97 ± 2.43 (n=6), respectively, p=0.0450] (Figure 2B).

Consistently, decreased engraftment resulted in a significant improvement in overall survival (OS) in animals transplanted with CRISPR 4E cells, specifically for MM6 cells OS was 48 days for CRISPR 4E (4EC6 n=10) vs 37 days for CTRL (CRTL7 n=10), p<0.0001 (Figure 2C). Similarly, for THP-1 CRISPR 4E (THP-1 4EC12, n=7) had an improved OS of 86 days compared with 59 days for THP-1 CRISPR-CTRL cells (THP-1 CTRL10; n=10), p=0.0002 (Figure 2C). The additional MM6 clone showed an OS of 51 days for CRISPR 4E (4EC1; n=5) vs 32.5 days for CTRL (CRTL13; n=4), p=0.0040 (Supplemental Figure 2C). Of note, animals injected with THP-1 CRISPR-CTRL cells all succumb with extramedullary tumors (abdomen) while only 2 animals from the THP-1 CRISPR 4E cohort presented small abdominal tumors (Supplemental Figure 3D), suggesting that the deficiency in eIF4E may have also prevented the cells from migrating to form abdominal tumors. Finally, we isolated human leukemic cells from the mice and examined eIF4E levels to ascertain whether cells retained their reduced levels of eIF4E (Supplemental Figures 2D and 3E). In summary when transplanted into mice, cells with reduced eIF4E levels which correspond to reduced levels of Ezrin, CD44, and HA (Figure 1A–B) were characterized by reduced engraftment with concomitant increased overall survival in these animals demonstrating a role for eIF4E in controlling these processes (Figures 1 and 2, Supplemental Figures 2 and 3).

In addition to the genetic manipulation of eIF4E, we studied pharmacological inhibition of eIF4E to test the impact on OS using primary AML cells as patient derived xenografts (PDXs). First, we compared the invasion capacity of high-eIF4E AML primary samples (AML55 and AML23) after ribavirin treatment *ex vivo*. We observed that both specimens had approximately 2–3-fold reduction in invasion after ribavirin treatment relative to vehicle treated cells (p= 0.0158 and 0.0123 respectively) (Figure 2D). Moreover, AML55, AML23 and another high-eIF4E sample AML13 had ~2–7-fold increased invasion relative to CD34+ cells from healthy donors (Supplemental Figure 4A). Thus, ribavirin treatment reduced AML55 and AML23 invasion nearing that to healthy donors (Figure 2D). To extend this into an *in vivo* model, we established an AML55-PDX mice which received two cycles of ribavirin (100mg/kg) (Figure 2E upper panel). Peripheral blood (PB) samples were obtained after the end of the second cycle (day111) where we found that animals treated with ribavirin had significantly less AML cells than mice treated with vehicle control (0.9% ± 0.24; n=5, vs. 7.5% ± 3.8; n=5, respectively, p=0.0079) (Supplemental Figure 4A and B). Approximately a week later, the fast progression of AML is observed in the Vehicle control (saline) mice when compared with the ribavirin treated group (44.9% ± 44.9; n=5) vs. 2.4% ± 1.65; n=5, respectively, p=0.0079] (Supplemental Figure 4B). Importantly, mice that received ribavirin survived ~51 days longer than the control animals treated with saline control (ribavirin cohort median survival=189 days vs saline=138 days; p=0.0384). Altogether, we demonstrated that the genetic depletion or pharmacological inhibition of eIF4E impairs engraftment within the BM niche and improves overall survival in AML *in vitro* and *in vivo* models and further support the importance of eIF4E for AML cell motility.

### A novel interplay between the motility and translation machineries

**Figure 3.**
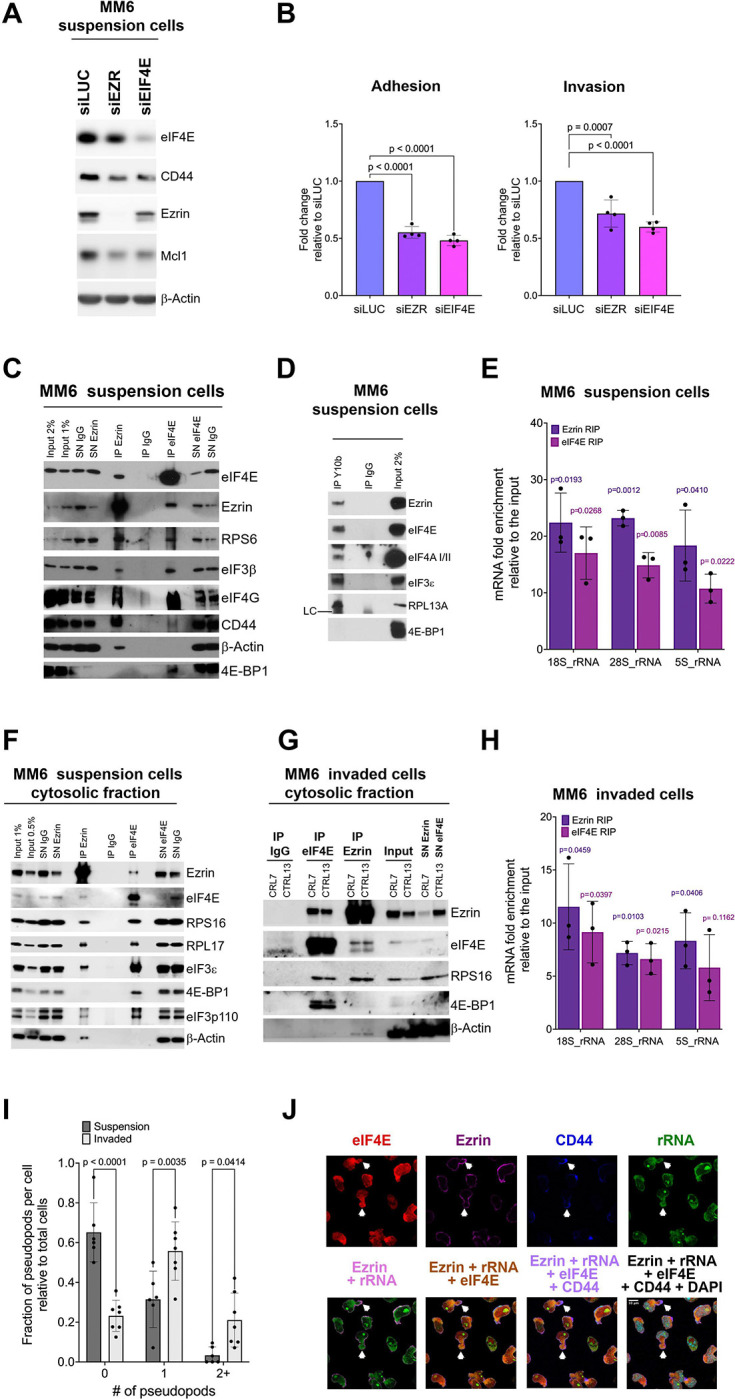
Ezrin and eIF4E functionally and physically interact. **A.** Western blot of total cell lysates from MM6 cells grown in suspension demonstrated knockdown of Ezrin (siEZR) or eIF4E (siEIF4E) compared to RNAi to luciferase (siLUC) used as a negative control. b-Actin is provided as a loading control. Other proteins of the Ezrin-CD44-HA axis are also shown. Quantification for these is shown in Supplemental Figure 5A with 3–6 biological replicates for each protein. **B.** Adhesion and invasion capacity of MM6 cells onto/through HS-5 bone marrow stroma. Fold change relative to siLUC is shown. Each symbol represents a biological replicate performed independently with replicates. Bars represent the mean, shown with standard deviations and p-values (one-way ANOVA). **C.** Western blot of eIF4E and Ezrin immunoprecipitations (IPs) using total cell lysates from MM6 cells in suspension. SN, supernatant after immunoprecipitation, IgG, negative control. Representative of three biological replicates. IPs of total cell lysates from THP-1 cells in suspension are provided in Supplemental Figure 5B. **D.** IPs from MM6 cells in suspension using the rRNA antibody Y10b. LC indicates antibody light chain. Representative of three biological replicates. **E.** RNA immunoprecipitations (RIPs) from MM6 total cell lysates grown in suspension using anti-Ezrin (Ezrin RIP) or anti-eIF4E (eIF4E RIP) antibodies. Data are from RT-qPCR and represented relative to input. Each symbol represents a biological replicate performed independently with triplicates. Bars represent the mean, shown with standard deviations and p-values (two tailed Welch’s t test). **F.** Western blots of eIF4E and Ezrin IPs from the cytoplasmic fractions of MM6 cells in suspension or **G**. After invasion through HS-5 bone marrow stroma (invaded). Fractionation controls are provided in Supplemental Figure 5D. **H.** RIPs from MM6 cytoplasmic fraction from invaded cells using anti-eIF4E (eIF4E RIP) or anti-Ezrin (Ezrin RIP) antibodies. Data are from RT-qPCR represented relative to input. Each symbol represents a biological replicate performed independently with triplicates. Bars represent the mean, shown with standard deviations and p-values (two tailed Welch’s t test). **I.** Count of the number of pseudopods observed in suspension and invaded MM6 cells represented as a fraction relative to the total cells counted. Each symbol represents a biological replicate. Bars represent the mean, shown with standard deviations and p-values (two-way ANOVA). **J**. Immunofluorescence and confocal microscopy demonstrating eIF4E, Ezrin, CD44 and rRNA are localized to the same pseudopods (white arrows). All confocal micrographs represent a single section through the plane of the cell. Scale bar = 10 μm

Given the role of eIF4E in motility shown above and the already known role of the Ezrin-CD44-HA circuit in AML, we assessed whether eIF4E-dependent motility required Ezrin. Here, we monitored the impact of genetic reduction on adhesion and invasion through HS-5 BM stroma cells using siRNA to *EZR* in MM6 cells which are characterized by high-eIF4E and compared to siRNA to luciferase (siLUC; which served as negative control). siRNA to *EIF4E* was used for comparison. RNAi reduced Ezrin levels by >10-fold relative to siLUC as observed by western blot with no impact on eIF4E protein levels (Figure 3A, Supplemental Figure 5A). Decreased Ezrin levels reduced adhesion by ~2-fold and invasion by ~1.5-fold relative to siLUC controls (Figure 3B). By comparison, eIF4E siRNA led to reduced levels of Ezrin and CD44 corresponding to a ~2-fold reduction relative to siLUC control (Supplemental Figure 5A), similar to the decreased levels of Ezrin and CD44 in the CRISPR 4E versus CRISPR-CTRL cells (Figure 1B) and subsequent ~3-fold reduction in CRISPR 4E versus CRISPR-CTRL MM6 cells (Figure 1D; Supplemental Figure 1D). We note that siEIF4E caused the anticipated reduction in CD44 and HA biosynthetic enzymes similar to CRIPSR 4E (Figure 1B). Interestingly, Ezrin caused similar changes which will be explored below. In all, Ezrin is required for eIF4E-dependent cell motility.

Above we showed that eIF4E controlled the production of the Ezrin-CD44-HA axis and that motility was Ezrin-dependent while Ezrin reduction did not affect eIF4E protein levels (Figure 3A, Supplemental Figure 5A). This strongly suggested a model whereby reduction in Ezrin impaired motility in AML cells. While this seems very likely, we also investigated whether an additional mechanism was also in play: Did Ezrin play an unanticipated physical role in translation? To address this, we first investigated whether there were physical interactions between eIF4E and Ezrin using immunoprecipitation of endogenous factors (Figure 3C–H; Supplemental Figure 5B). MM6 and THP-1 cells were crosslinked immediately prior to collection to avoid reassortment during processing and immunoprecipitation. We observed that endogenous eIF4E immunoprecipitated with Ezrin and CD44 in total cell lysates from both cell lines as well as in cytoplasmic fractions in MM6 cells (Figure 3C, F and Supplemental Figure 5B). Conversely, endogenous Ezrin immunoprecipitated with eIF4E as well as CD44 and Actin in total cell lysates and from cytoplasmic fractions (Figure 3C, F, Supplemental Figure 5B and 5C). b-Actin served as a negative control for eIF4E, and 4E-BP1 for Ezrin immunoprecipitations; and moreover, suggested that eIF4E interacts with a form of Ezrin that is not Actin associated. Factors were not observed in the IgG negative controls.

Next, we assessed whether other components of the translation machinery also interacted with Ezrin (Figure 3C–H; Supplemental Figure 5B and C). Strikingly, endogenous Ezrin interacted with several key components of the translation machinery including eIF4E, translation initiation factors eIF4AI/III, eIF3 components (3b, 3e, 3p110), and the ribosomal small and large subunit proteins RPS6, RPS16 and RPL17. Endogenous eIF4E interacted with these factors as expected. Interestingly, eIF4E also immunoprecipitated with CD44, which is known to directly bind to Ezrin at the plasma membrane^40^ (Figure 3C). As expected, Ezrin did not immunoprecipitate with 4E-BP1 a negative regulator of eIF4E. By contrast, eIF4E immunoprecipitated with 4E-BP1 as expected, consistent with eIF4E immunoprecipitations isolating both translationally active and inactive forms of eIF4E. We confirmed the interactions of Ezrin with eIF4E and the translation machinery in THP-1 cells (Supplemental Figure 5B). To explore if Ezrin is associated with intact ribosomes, we carried out RNA immunoprecipitation (RIP) and RT-qPCR monitoring interactions with ribosomal rRNAs (5S, 18S and 28S rRNAs) from total cell lysates of MM6 cells. Indeed, Ezrin RIPs were characterized by an 18–22-fold enrichment in rRNAs over input in MM6 cells (Figure 3E). For comparison, eIF4E RIPs were 10–17-fold enriched for these rRNAs. Minor differences in fold changes between Ezrin and eIF4E RIPs likely arise due to differences in avidity of the antibodies. To independently validate the physical association of Ezrin with ribosomes, we employed the Y10b antibody to rRNAs^50^. Using crosslinked total cell lysates, we observed that Y10b immunoprecipitated Ezrin as well as positive controls eIF4E and the translation machinery but not the 4E-BP1 (Figure 3D).

Given the considerable structural rearrangements in cells upon invasion including the formation of pseudopods, we monitored interactions of these factors in invaded cells. Here after invasion, we fixed MM6 cells from the lower compartment of the Boyden chamber, collected these and monitored whether Ezrin interactions were preserved. With this material, we conducted immunoprecipitation experiments from cytoplasmic fractions from invaded cells (Figure 3G–H). We note that there is substantial reduction in invaded cell number relative to cells grown in suspension yielding only about 100 μg of material which limited the extent of immunoprecipitation analyses. We observed that eIF4E and Ezrin co-immunoprecipitated and both associated with RPS16 protein. Ezrin did not bind 4E-BP1 while eIF4E did associate with 4E-BP1 consistent with findings in suspension cells. Moreover, both Ezrin and eIF4E immunoprecipitated with rRNAs relative to input 5–16-fold (Figure 3H). Thus, Ezrin binds ribosomes in invaded cells. MEK, CalR and Nopp140, H3K27A were used as cytoplasmic and nuclear markers respectively to show the quality of the fractionation (Supplemental Figure 5D). Thus, the interaction of Ezrin with the translation machinery is preserved in invaded cells. In all, we show interactions between Ezrin, eIF4E and the translation machinery in multiple cellular contexts suggesting that Ezrin plays an important role in specialized translation likely to produce factors required for motility.

### Pseudopods in motile AML cells are sites of active translation

Given the relevance of pseudopods to AML cell motility and the association of CD44 and Ezrin with such structures, we set out to assess if eIF4E could be playing a role in a novel form of translation there. To assess the best conditions to monitor pseudopods, we set out to quantify the difference in pseudopod numbers per AML cell before and after HS-5 BM stroma invasion. We observed most invaded cells had at least one pseudopod. Indeed, ~80% of invaded compared to ~35% of suspension cells exhibited pseudopods (Figure 3I). Given the higher prevalence of pseudopods in invaded cells, we focused on these for the remaining studies unless otherwise noted. Taken together with the immunoprecipitation, our data suggest that these pseudopods are sites of active translation or storage depots for this machinery.

To examine this possibility, we first assessed the spatial localization of eIF4E and ribosomal rRNAs relative to Ezrin and CD44 employing immunofluorescence and confocal microscopy with Z-stacks (Figure 3J). To capture invaded cells, cells in the lower compartment of the Boyden chamber after invasion through HS-5 cells were fixed with paraformaldehyde (PFA) *in situ*, collected and then underwent processing for immunofluorescence to assess the localization of eIF4E, Ezrin, rRNA (Y10b antibody) and DAPI as a nuclear marker (Figure 3J). To ensure there were no physical or optical interference, each wavelength was collected independently, and single staining controls showed no reorganization or bleed through of signal between single and multiple staining experiments (data not shown). In MM6 cell in suspension, eIF4E is found in nuclear bodies as well as in the cytoplasm as reported^51–54^ (Supplemental Figures 1B). In invaded MM6 cells substantial fraction of eIF4E is, interestingly, located in pseudopods where the majority of Ezrin and CD44 are found (Figure 3J). We observed a highly similar pattern for eIF4E localization in invaded relative to suspension cells with both characterized by nuclear eIF4E and cytoplasmic eIF4E, as well as an eIF4E-enrichment in pseudopods in these cells (Figure 3J). As expected, rRNAs were also found in the nucleolus. Thus, consistent with the immunoprecipitation data (Figure 3 C–H), we observed that Ezrin was localized to the pseudopods containing eIF4E, rRNA and CD44 (Figure 3J).

### Pseudopods are sites of active translation

**Figure 4.**
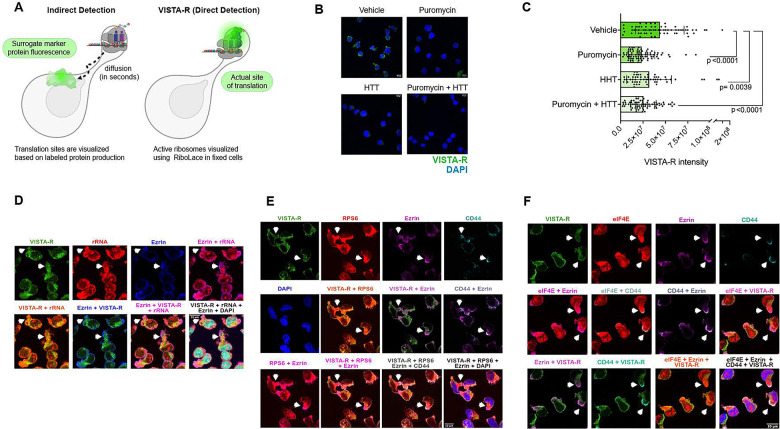
Active ribosomes and translation machinery are observed in T-PODs. **A**. Schematic comparing previous strategies to monitor in situ translation based on surrogate markers of translation such as labelled amino acids (left), and our novel method for direct measurement of translation, VISTA-R (right). **B.** VISTA-R signals in MM6 cells are directly related to translation activity since these are abrogated upon addition of translation inhibitors. Puromycin and/or HHT were added prior to the fixation step in VISTA-R. Representative confocal micrograph of VISTA-R signal in vehicle control, puromycin, HHT or combination treated cells. DAPI is shown in blue. Scale bar = 10 μm. **C.** Quantitation showing means, standard deviations of the mean, data points from each measurement, p-values calculated with one way ANOVA test from over 60 cells. **D.** T-PODs (as shown by arrows) contain active ribosomes as seen by VISTA-R (green). VISTA-R and rRNA (red), Ezrin (blue) overlay in these T-POD. **E.** Same as D but demonstrating VISTA-R (green), Ezrin (magenta) and RPS6 (red) and CD44 (turquoise) are present in the same T-PODs (see arrows). **F.** Same as D-E but demonstrating that VISTA-R (green), eIF4E (red) and Ezrin (magenta) and CD44 (turquoise) are present in the same T-PODs (see arrows). All confocal micrographs represent a single section through the plane of the cell. Scale bars = 10 μm.

Next, we sought to ascertain whether the pseudopods were sites of active ribosomes or storage sites for translation factors. To this end, we developed a method to directly mark active ribosomes. Several limitations with existing methods were overcome to mark active ribosomes *in situ*. Foremost is that proteins diffuse from the site of protein synthesis within seconds^55^. Thus, the use of labelled amino acids to mark sites of translation is problematic since the newly synthesized fluorescent proteins rapidly diffuse from ribosomes^55^ (Figure 4A, left panel). This clearly confounds determination of the actual sites of translation. To overcome this, we developed a new assay referred to as Visualizing Translation Activity using RiboLace (VISTA-R). VISTA-R is an *in situ* translation assay which employs the biotinylated puromycin analogue, RiboLace, which binds directly to active ribosomes^56^ (Figure 4A right panel, Supplemental Figure 6A). RiboLace, like puromycin, enters the empty A-site of the active ribosome mimicking a tRNA, however this leads to premature chain termination, release of the peptide, and ribosomal dissociation^56^. To overcome these issues, cells were fixed with PFA to freeze ribosomes prior to RiboLace treatment. Then RiboLace signal was subsequently detected by streptavidin-fluorophore conjugate and confocal microscopy. In this scenario, we are not monitoring the incorporation of RiboLace into the peptide chain but rather detecting its presence on the active ribosome. Using confocal microscopy, we observed RiboLace signals throughout most of the cytoplasm, and importantly, there was no nuclear signal detected consistent with the absence of diffusion using this strategy (Figure 4B,D–F). To confirm RiboLace staining was marking translation, we inhibited translation elongation using puromycin (30 minutes at 250μg/mL) to allow the ribosomes to dissociate and then fixed and followed the VISTA-R protocol and quantified in Imaris software. Here, we monitored signal across the entire cell volume and observed a ~2-fold reduction in signal (green) upon puromycin treatment consistent with the VISTA-R method detecting active translation (Figure 4C). We observed similar results using the translation inhibitor homoharringtonine (HHT) (30 minutes, 10μM) which also binds the A-site but stalls translation initiation^57^. The VISTA-R signal resulted from treatment with a combination of HHT and puromycin for 30 minutes was also ~2-fold reduction. These are strong reductions in VISTA-R signals considering it was a 30 minute treatment (Figure 4C). We note that complete abrogation of signal would not be expected here given that along with the inhibitor is competition between reinitiation of translation after inhibition. For these experiments, we note that we used suspension rather than invaded cells given the lower number of cells that could be obtained the invasion experiments (Figure 4B). In all, VISTA-R allows detection of active ribosomes *in situ*.

We combined VISTA-R with immunofluorescence to monitor if Ezrin-positive pseudopods were sites of translation using spinning disk confocal microscopy (Figure 4D–F). In invaded cells, pseudopods contained VISTA-R and rRNAs which overlapped (in orange, see arrows, Figure 4D) again indicative of active translation occurring in these structures and further confirming the VISTA-R is marking active ribosomes. VISTA-R, Ezrin, and CD44 were also found in the same pseudopods where they overlapped (Figure 4E in white, see arrows). Consistently, RPS6 and Ezrin partially overlapped in the same pseudopods. As expected, eIF4E was also found in these translationally active pseudopods and overlaid with Ezrin particularly at the tips of these structures (Figure 4E). In terms of structural organization, eIF4E and active ribosomes tended to be more internally localized while Ezrin and CD44 tended to be more enriched around the periphery of these structures (Figure 4D–F). Finally, primary AML patient specimens displayed similar translational activity Ezrin-positive pseudopods indicative of the clinical relevance of these structures (Supplemental Figure 6B). Given that we observed eIF4E, RPS6, rRNA and active ribosomes (via VISTA-R) overlaid in the same pseudopods, we refer to these structures as translationally active pseudopods (T-PODs).

### Translation in T-PODs is Ezrin- and eIF4E-dependent

**Figure 5.**
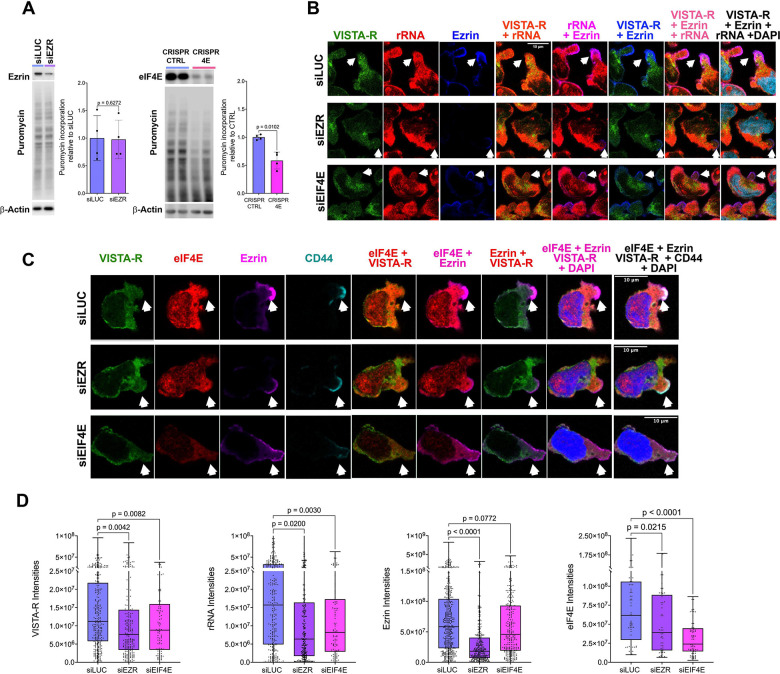
Ezrin influences localized translation in T-PODs but not global translation. **A.** Western blots of total cell lysates for MM6 cells in suspension treated with puromycin and analyzed by WB with anti-puromycin antibody (to assess global protein synthesis) reveal that Ezrin does not influence global translation: RNAi knockdown to Ezrin (siEZR) did not show difference compared to luciferase (siLUC) control (left panel). In contrast, CRISPR 4E reduces global translation signal by about 50% relative to CRISPR-CTRL, consistent with previous studies. Each symbol represents a biological replicate, bar showing mean, standard deviation, p-value (two tailed paired t test). **B.** Confocal micrographs for VISTA-R and the indicated proteins for MM6 cells upon genetic knockdowns siEIF4E), siEZR or siLUC control Arrows indicate T-PODs. Scale bar = 10μm. Western blots confirmed knockdown (Supplementary Figure 6C) **C.** As in B but monitoring eIF4E in T-PODs as a function of Ezrin and eIF4E knockdown. Ezrin is also reduced by eIF4E knockdown in invaded cells (Supplementary Figure 6C). Scale bar = 10μm. **D.** Quantitation of intensity of factors as noted in T-PODs over 3 biological replicates for VISTA-R and rRNA, 5 for Ezrin and 2 for eIF4E. Boxplots show Imaris-quantified TPOD-associated intensities of VISTA-R, rRNA, Ezrin, and eIF4E under the conditions shown. Each dot represents an individual T-POD object, p-values calculated using one way ANOVA test.

We next determined the role of Ezrin in translation. We anticipated that Ezrin would not influence global translation given its enrichment in pseudopods in invaded and suspension cells. Given the material required for global protein assays, these studies were carried out on suspension rather than invaded cells. Here, we observed that Ezrin knockdown using siRNA did not influence global protein production when compared to negative control knockdown to luciferase (siLUC) by western blot (Figure 5A left panel). For comparison, eIF4E reduction using MM6 CRISPR 4E versus MM6 CRISPR-CTRL cell lines demonstrated reduced translation by ~50% similar to previous studies using eIF4E antisense oligonucleotides which reduced global translation by ~50%^58^ (Figure 5A, right panel). These latter findings are consistent with the observation that eIF4E affects the translation of many, but not all, transcripts. In all, genetic reduction in Ezrin levels did not influence global translation consistent with its limited localization.

Next, we determined whether Ezrin influenced localized translation in T-PODs using invaded cells. To do this, siRNA knockdown of Ezrin and eIF4E were carried and compared to the negative control siLUC, knockdowns were validated by western blot (Supplemental Figure 6C). Confocal microscopy was used to monitor localization in T-PODs for active ribosomes (VISTA-R), rRNA (Y10b), Ezrin and DAPI (Figure 5B) and VISTA-R, eIF4E, Ezrin, CD44 and DAPI (Figure 5C). Arrows indicate example T-PODs. In siLUC controls, we observed similar results as for staining in parental invaded MM6 cells (Figure 5B–C, top row; Figure 4D–F), with Ezrin, eIF4E, rRNA and VISTA-R observed in the same pseudopods (Figure 5B–C, top row). Thus, siLUC treatment did not disrupt the localization of these factors or the translation activity in the T-PODs relative to invaded MM6 cells. By contrast, siEIF4E and siEZR treatment led to a reduction of these factors in T-PODs when compared to siLUC (Figure 5B–C middle and bottom rows).

To quantify the intensity in T-PODs for each channel, we used Imaris software with the following steps: 1. A surface corresponding to the nucleus was generated using the DAPI signal, 2. The MATLAB XTension *Distance Transformation* was applied to the DAPI-defined surface to generate a new surface capturing channel signals on and within the cellular membrane of each cell (T-PODs remained outside of this surface), 3. All protein signals within the Distance Transformation surface were removed by masking, 4. A new surface corresponding to T-PODs was generated using the newly created channel following masking of the Distance Transformation surface, 5. Signal intensities for each channel were measured within the T-POD surfaces (Supplemental Figure 6D). Provided calculated intensities are per T-POD. Using this strategy, we observed that eIF4E knockdown reduced the VISTA-R signal and rRNA intensities by 1.5- and 1.9-fold per T-POD relative to siLUC controls, respectively (Figure 5D). eIF4E knockdown also reduced Ezrin levels per T-POD (Figure 5D) consistent with eIF4E reducing Ezrin protein levels in total cell lysates (Figure 1B, 3A, Supplemental Figure 5A. Supplemental Figure 6C). Interestingly, Ezrin knockdown reduced the signal per-T-POD from active ribosomes (VISTA-R) 1.5-fold and rRNA 2.3-fold relative to siLUC controls (Figure 5B–D). For positive controls, signals were reduced for eIF4E (2-fold) and Ezrin (2.5-fold) per-T-POD upon their respective knockdowns (Figure 5D). Importantly, although rRNA levels were reduced per TPODs upon Ezrin or eIF4E knockdown (Figure 5B–D), their overall levels in cells were not altered by RT-qPCR (Supplemental 6E). Given the following findings: (1) reduction in Ezrin decreased levels of rRNA, active ribosomes and eIF4E per T-POD (Figure 5B–D), (2) Ezrin did not impact global translation, or total levels of eIF4E or rRNA (Figure 5A; Supplemental Figure 6C&E), (3) Ezrin binds the transmembrane protein CD44 (Figure 3C)^59^, and (4) Ezrin’s enrichment in T-PODs (Figures 3L, 4C–E, 5B–D); we conclude that the role of Ezrin is to anchor the active ribosomes to T-PODs. Whether this anchoring is direct or through other factors is yet to be determined. Consistently, we show that translation in T-PODs is dependent on both Ezrin and eIF4E.

### Ezrin physically associated with transcripts encoding motility factors and impacted their protein production

While VISTA-R enabled us to observe active ribosomes in T-PODs in fixed cells, we recognized that identifying actual targets of T-POD translation would be challenging. The following issues to identify transcripts that undergo Ezrin-dependent translation needed to be considered in order to develop fruitful solutions: (1) isolation of protrusions after treatment with labelled amino acids and followed by protein analysis would not reliably identify newly synthesized proteins generated in T-PODs given rapid diffusion of protein products from ribosomes (in seconds) and diffusion between T-PODs and bulk cytosol which would confound assignment of the origin of translation, (2) measurement of translation of bulk translational efficiency using polysomes or ribosome foot-printing methods would measure translation efficiency over the entire cell whereby T-POD translation is a small (but physiologically important) fraction which would not be detected over the bulk signal, and (3) in invaded cells or in isolated protrusions there is not sufficient material to measure translation efficiency. Thus, we opted, as a first step, to identify RNA targets of Ezrin-dependent translation which should allow us to identify T-POD translation targets by leveraging the enrichment of Ezrin in these structures in invaded cells and the physical association between Ezrin and the translation machinery (Figure 3).

**Figure 6.**
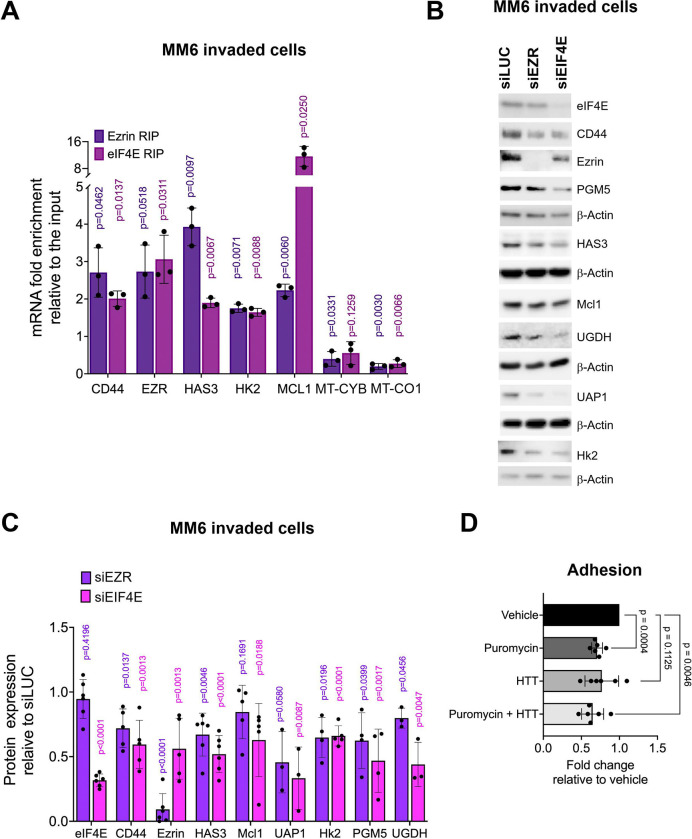
Ezrin physically associates with RNAs encoding motility factors and influences their protein production. **A.** Ezrin and eIF4E RIPs from cytoplasmic fractions from invaded MM6 cells compared to input. Each symbol represents a biological replicate. Means, standard deviations, p-values (Welch’s t test) are shown. Mitochondrial encoded mRNAs were used as negative controls (MT-CYB and MT-CO1). **B.** Representative western blots showing the expression for the indicated proteins using total cell lysates of invaded MM6 cells with knockdown of Ezrin (siEZR) or eIF4E (siEIF4E) and siLUC control. Quantitation of these western blots shown in **C**, each symbol represents a biological replicate, bar shows the mean, standard deviations and p-values (multiple unpaired t tests). **D.** Translation inhibitors decrease the adhesion capacity of MM6 cells. Each dot represents a biological replicate. Means, standard deviations and p-values (one way ANOVA) are shown.

With this in mind, we fixed invaded cells in the lower Boyden chamber, collected these, isolated the cytoplasmic fraction which then underwent immunoprecipitations with Ezrin or eIF4E antibodies or the negative control IgG. Biological replicates were conducted on parental MM6 or MM6-CTRL cell lines (Figure 6A). Results were compared to eIF4E RIPs as shared targets would provide strong evidence that these are indeed translation targets. Cytoplasmic fractions were used to avoid isolating eIF4E-dependent mRNA export targets from the nucleus; the quality of the fractions is shown (Supplemental Figure 5D). Only very little material was isolated from cytoplasmic fractions of these invaded cells. Indeed, we isolated ~100 μg of material per chamber which then underwent fractionation and immunoprecipitation and thus not all mRNAs were detected in the cytoplasmic input due to these low amounts of material. We also note that in some cases no signal was detected in the IgG negative control by RTqPCR, which confirms no background binding, but these obviously could not be plotted. We observed that Ezrin RIPs with transcripts corresponding to the Ezrin-CD44-HA pathway including *CD44, Ezrin* and transcripts encoding factors involved in HA biosynthesis *HAS3*, and *HK2* by 2–4-fold relative to input (Figure 6A). We reasoned that Ezrin RIP targets that were translation targets would also physically associate with eIF4E. Consistent with this hypothesis, eIF4E RIPs with these same targets by ~2-fold relative to input (Figure 6A). As negative controls, we examined eIF4E and Ezrin RIPs of mitochondrially encoded transcripts cytochrome B (*MT-CYB*) and cytochrome oxidase 1 (*MT-CO1*), consistently neither was enriched in eIF4E or Ezrin RIPs. Interestingly, MCL1 was much more enriched in eIF4E RIPs (12-fold relative to input) consistent with eIF4E’s presence in both the cytoplasm (bulk translation) and T-PODs while Ezrin RIPs were considerably less (2-fold versus input) consistent with its enrichment in T-PODs but not in the cytoplasm. We ruled out effects of Ezrin or eIF4E on mRNA stability and/or transcription of these target transcripts: we observed no alteration in mRNA levels between siEZR or siEIF4E relative to siLUC treatments on mRNAs isolated from total cell lysates derived from invaded cells (Supplemental Figure 6E). Moreover, the knockdowns did not impact the total levels of 18S, 28S or 5S rRNAs (Supplemental figure 6E) suggesting that the number of ribosomes were not influenced.

Thus, Ezrin and eIF4E did not influence the steady-state stability or transcription of these transcripts; and moreover, Ezrin which is substantially enriched in T-PODs is physically associated with these mRNAs. In all, these findings position Ezrin as a direct modulator of the translation of factors in the Ezrin-CD44-HA axis through physical association of Ezrin with the corresponding target mRNAs.

Next, we sought to determine if Ezrin influenced protein production of these targets (Figure 6B and C). Given the issues described above related to the rapid diffusion of newly synthesized proteins from sites of translation and the diffusion between T-PODs and the cytosol, we did not isolate T-PODs. Rather we opted to measure total protein lysates by western blot and establish whether modulation of Ezrin incurred significant changes in the levels of these factors despite the relatively low level of T-POD translation compared to cytoplasmic translation. Here, invaded cells were collected and total cell lysates prepared. We monitored the impact of knockdown of Ezrin compared to siLUC and with knockdown of eIF4E as a positive control. Despite the limitations, siEZR decreased protein levels by ~40–60% for CD44, HAS3, HK2, PGM5, and UAP1 proteins and around ~25% for UGDH compared to siLUC (Figure 6C). MCL1 trended to ~20% reduction upon siEZR relative to siLUC controls without reaching statistical significance. Importantly, reduction in Ezrin did not affect eIF4E protein levels or the negative controls b-Actin (Figure 6B). We anticipated greater effects for eIF4E than Ezrin on target protein production given eIF4E plays a direct role in the nuclear export of these target mRNAs thereby increasing their availability to the ribosome^45^, and that eIF4E is found throughout the cytoplasm and thus can influence translation outside of T-PODs as well as the capacity of eIF4E to reduce Ezrin protein levels themselves by ~50%. Indeed, genetic knockdown of eIF4E reduced protein levels for CD44, HAS3, HK2, UAP1, PGM5, UGDH and MCL1 by ~40–70% in invaded cells relative to siLUC controls (Figure 6C). Similar findings were observed with CRISPR mediated knockdown of eIF4E in invaded cells and in cells grown in suspension (Figure 1B, Supplemental Figure 1D, Supplemental Figure 6F) demonstrating the very robust impact of eIF4E on these pathways. In all, impact of Ezrin and eIF4E were highly similar on protein production of the Ezrin-CD44-HA axis but with eIF4E knockdown consistently causing greater reductions as expected. There is a strong correlation between the mRNA targets Ezrin and eIF4E immunoprecipitated in the cytoplasmic fraction of and the effect on corresponding protein production. Taken together with our findings that Ezrin physically associated with rRNAs, ribosomal proteins, translation initiation factors, mRNAs encoding the motility circuit, and that Ezrin and eIF4E overlap with active ribosomes in T-PODs, our studies revealed a role for Ezrin in modulation of translation of specific mRNAs encoding motility factors in the Ezrin-CD44-HA circuit.

### Translation activity is required for preservation of pseudopods and AML cell motility

Next, we hypothesized that if high-eIF4E AML cell motility were translation-dependent, it should be impaired by translation inhibitors. We examined adhesion of MM6 cells onto HS-5 stroma using MM6 parental and MM6 CRISPR-CTRL cells for biological replicates. Invasion assays were not conducted as puromycin and HHT severely reduced cell viability after 24 hours, the timeframe of the assays; but adhesion assays were possible given viability was not impacted in this 30 minute timeframe (Figure 6D). We found that puromycin inhibited adhesion by ~30%, with a similar trend for HHT, substantial changes given the short treatments. Addition of both inhibitors further decreased adhesion by ~40% relative to vehicle controls. This is consistent with our observation that genetic or pharmacological inhibition of eIF4E impairs AML cell motility in our PDX and AML patient models (Figure 2). In all, our studies reveal a novel translation program modulated by the motility machinery underpinning AML cell movement.

## Discussion

A major reason for cancer treatment failure is an inability to halt cancer cell motility leading to widespread metastatic disease. In AML, leukemic cell motility is central to disease progression, treatment resistance and disease relapse, as they actively migrate between microenvironmental niches. Thus, elucidating the mechanism controlling this process of migration is instrumental to identifying new therapeutic targets. Here, we provide novel insights into cell movement demonstrating that AML cell motility *in vivo* and *in vitro* was driven at the post-transcriptional level by eIF4E providing an important addition to the overarching mechanisms underpinning these processes. Indeed, these are the first studies in mouse models to show that eIF4E played a key role in leukemogenesis. Modulation of eIF4E alone impacted adhesion, invasion and colonization *ex vivo* using AML cell line models and patient primary specimens; and also affects engraftment and leukemia burden *in vivo* using CDX and PDX models (Figures 1–2, Supplemental Figure 2–4). The two CDX models (MM6 and THP-1 CRISPR cell lines) we developed demonstrated a strong reliance on eIF4E for BM engraftment and a robust relationship between a reduction in eIF4E levels and improving overall survival (Figure 1–2, Supplemental Figures 2–4). This corresponded to eIF4E-dependent targeting of the Ezrin-CD44-HA circuit. These findings are consistent with previous observations that elevated eIF4E alone predicted poor outcomes in AML patients (Leucegene.ca)^9^. Using pharmacological inhibition of eIF4E with ribavirin in PDX mouse models we reproduced the genetic disruption of eIF4E observations in CDX models. Furthermore, these was consistent with our 3D spheroid model that mimics the BM niche. Suggesting that these models can be leveraged to assess the impact of targeting Ezrin and other components of the Ezrin-CD44-HA circuit in AML to develop new therapies in combination with eIF4E inhibitors such as ribavirin. Indeed, CD44 and HA have been targeted in clinical trials^60–62^ with positive outcomes and serve as important directions for future therapeutic development while Ezrin has not yet been targeted in the clinic.

Dissection of the underlying molecular mechanisms by which eIF4E drove motility and disease progression revealed two important findings: (1) a novel interplay between the motility and translation machinery and (2) localized translation in pseudopods structures that are directly related to AML ameboid movement. Specifically, we demonstrated that Ezrin was required for eIF4E-dependent AML cell motility providing a functional linkage between the translation and cell motility machineries. This functional linkage is paralleled by the physical association of Ezrin with eIF4E, the translation machinery, active ribosomes and transcripts encoding motility factors including CD44. Ezrin is mainly found in T-PODs in invaded cells which we show contain active ribosomes, eIF4E and rRNAs. Reduction of Ezrin causes: (1) reduced association of rRNAs, active ribosomes and eIF4E at the T-PODs implicating Ezrin as an anchor for T-POD localized translation, (2) decreased protein production of components of the Ezrin-CD44-HA axis including CD44, with no change to steady-state transcription or stability of the corresponding mRNAs indicative of a role for Ezrin in translational control and (3) impaired eIF4E-dependent AML cell adhesion and BM invasion. Indeed, this study revealed for the first time that Ezrin modulated the production of any protein, much less one so closely related to its traditional function in transducing HA signals to Actin via CD44. In relation to this function, the N-terminal domain of Ezrin directly interacts with the intracellular C-terminal tail of CD44, while it utilizes its C-terminal domain to bind to Actin, which permits the physical deformations related to cell locomotion^39,63^. Presumably, Ezrin anchors the active ribosomes, mRNAs, eIF4E and rRNAs in T-PODs via its direct interactions with CD44 which is anchored to the membrane *via* its transmembrane protein. The physical organization of eIF4E and Ezrin within the T-PODs and the precise role that Ezrin plays in translation are important areas of future study. In all, we demonstrate that Ezrin plays a role in the translation of motility factors and that this occurs in T-PODs likely to provision oncogenic migration.

The discovery of T-PODs relied on our development of VISTA-R to overcome current methodological limitations in the study of localized translation. Prior to this, identification of translation sites relied on a surrogate for active ribosomes: detection of newly synthesized proteins using labelled amino acids. However, proteins quickly diffuse from active ribosomes leading to misassignment of translation sites^55^ (Figure 4A). To overcome this challenge, we directly monitored translation using VISTA-R which permitted us to observe active ribosomes in pseudopods *in situ*. Moreover, it enabled us to establish that translation within T-PODs relied on Ezrin and eIF4E. VISTA-R is readily adaptable to a wide array of systems providing an important new tool in the study of localized translation. The advantage of localized translation in T-PODs is that the delivery of mRNAs and ribosomes to sites of action enables multiple rounds of protein production on-demand. By contrast, delivery of the protein-of-interest to T-PODs (after cytoplasmic translation) would lead to rapid exhaustion of factors requiring constant re-supply. Generally, localized translation is expected to provide higher localized concentrations of proteins at their site-of-action than occurs when trafficking single proteins to motility sites from translation in the cytoplasm.

Taking into account our studies in the context of previous work, there are multifactorial effects of eIF4E on the Ezrin-CD44-HA circuit and resultant cell motility. Previous studies indicated that eIF4E increased the nuclear export of specific oncogenic mRNAs by ~2–10 fold including those in the Ezrin-CD44-HA axis and that this export activity contributed substantially to factor production as well as oncogenic potential^45,53,64–67^. Total levels of these RNAs were not impacted by decreased eIF4E indicating that eIF4E did not influence transcription or mRNA stability of these factors, as we observed here. We show here that additionally to these activities, eIF4E also drives translation in T-PODs where its reduction leads to decreased active ribosomes there and moreover, eIF4E physically interacted with both Ezrin and CD44 factors almost exclusively found in T-PODs in invaded cells (Figures 3–5). Indeed, Ezrin appears to anchor eIF4E, ribosome components and active ribosomes in T-PODs as shown by reduction of those factors upon genetic reduction of Ezrin (Figure 5). Notably, there is no Ezrin detected in the nuclei of AML cells at steady-state indicating that Ezrin does not play a role in eIF4E-dependent mRNA export (Figures 3–5). Taken together with these results, our findings suggest that in the nucleus eIF4E drives export of a specific subset of mRNAs encoding factors in the Ezrin-CD44-HA axis which are subsequently transported to T-PODs where they undergo eIF4E- and Ezrin-dependent translation to sustain motility. In this way, eIF4E acts to stimulate the Ezrin-CD44-HA circuit using multiple mechanisms including the novel physical interactions between the motility and translation machinery we discovered here. Given the physical interaction between eIF4E and Ezrin, it will be important in future studies to identify Ezrin-dependent USER codes to understand mRNA selection in localized translation. Given the high level of structural conservation amongst Ezrin and other FERM domain proteins including radixin and moesin^43^, these latter factors may also play similar roles in localized translation which should be studied in the future.

In conclusion, we discovered that eIF4E through the Ezrin-CD44-HA axis is a driver of AML, identified novel functions for Ezrin in translation, developed VISTA-R to mark active ribosomes *in situ* which led to the identification of a novel on-demand localized translation system (T-PODs) for rapid provisioning of factors at their sites-of-action to sustain movement.

### Limitations of the Study.

Biochemical studies to measure translation efficiency are not feasible when studying translation in pseudopods; and moreover, the low numbers of invaded cells only exacerbated this challenge. While polysome analysis using sucrose gradients is the gold standard in the field, and we have substantial experience with this methodology, it does not directly measure translation in T-PODs but rather measures bulk translation in the cytoplasm. Moreover, this method requires 1–5 million cells which is not achievable for invaded cells. While pseudopods can be isolated, the limited material presents an even greater problem in terms of measuring translation efficiency by polysomes. Ribosomal foot-printing methods require less material than polysomes but they measure ribosomal binding without a direct readout of whether such binding corresponds to increased translation efficiency. Again, such methods would need to be on pseudopods rather than cytoplasmic fractions and would need to take into account diffusion between the cytoplasm and T-PODs. Thus like many other groups that have faced these same challenges studying localized translation^68,69^, we directly measured production of the target proteins. However, we extended this by choosing targets whereby the corresponding mRNAs were bound to Ezrin and eIF4E bridging a direct link between target binding and corresponding protein production. In future, the development of more sensitive detection methods for translation efficiency coupled with ones that can be conducted *in situ* will make direct assessment of ribosome per transcripts in pseudopods feasible.

## Materials and Methods

### Cell lines and primary samples.

HS-5 cell line was obtained from the American Type Culture Collection (ATCC). MM6, THP-1, and NOMO-1 cell lines from Leibniz Institute DSMZ-German collection of microorganisms and cell cultures. All cell lines were authenticated and routinely tested for mycoplasma (PlasmoTest^™^, mycoplasma detection kit, rep-pt1, InvivoGen). HS-5 cells were cultured in Dulbecco’s Modified Eagle’s Medium (DMEM, cat# 11995–065, Gibco) with 10% fetal bovine serum (FBS, cat# 100–106, GeminiBio) and 1% penicillin-streptomycin solution (pen/strep, cat# 30–002, Corning); MM6, THP-1 and NOMO-1 cells were cultured in Roswell Park Memorial Institute medium (RPMI 1640, cat# 11875–093, Gibco) supplemented with 10% FBS and 1% pen/strep. MM6 cells were also supplemented with non-essential amino acids (cat# 11140–050, Gibco), 1 mM sodium pyruvate (cat# 11360–070, Gibco) and 10 μg/ml human insulin (cat# I9278, Sigma-Aldrich).

For eIF4E or Ezrin knockdowns, MM6 cells were transfected with corresponding siRNA duplexes using SF Cell Line 4D X Kit (Lonza) following program Jurkat on the Amaxa^®^ Nucleofector^®^ Technology (Amaxa Biosystems, Berlin, Germany). All siRNA were from IDT Technologies. siRNA duplex_Luciferase sense (CACGUACGCGGAAUACUUCGAAATG) and antisense (CAUUUCGAAGUAUUCCGCGUACGUGUU) siRNA duplex _Ezrin sense (GGAAUCAACUAUUUCGAG) and antisense (UUUUUAUCUCGAAAUAGU); siRNA duplex eIF4E: sense (CUGCGUCAAGCAAUCGAGAUUUGGG) and antisense (CCCAAAUCUCGAUUGCUUGACGCAGUC).

MM6 and THP-1 CRISPR clones with eIF4E disruption were generated using Lenticrisprv2 plasmid purchased from Addgene^70,71^. Lenticrisprv2 plasmids with sgRNA inserts (EIF4E: TTAAACATCCCCTACAGAAC, and Azami-Green protein as the control: GGCCACAACTTCGTGATCGA) were kindly provided by the lab of Dr. Mike Tyers. MM6 cells (ATCC) were transduced using lentiviral particles containing these plasmids and produced in 293T cells and selected with puromycin at 1μg/ml. Clones were screened for eIF4E expression by western blot. Knockdown in clones with reduced protein expression was confirmed by Sanger sequencing.

NOMO-1 overexpressing cell lines were produced by retroviral transduction. Briefly, eIF4E wild-type retroviral vectors along with pCL-Ampho packaging vector were transiently transfected into 293T cells (ATCC), and retroviral supernatants were used to infect NOMO-1 cells. GFP^+^ cells were isolated using a cell sorter (FACSAria; BD Biosciences).

There were three sources for deidentified primary human specimens all of which were obtained with written informed consent according to the Declaration of Helsinki an IRB/Ethics Committee institutional approvals: Weill Cornell Medicine, Banque de cellules leucémiques du Québec (BCLQ), supported by the Cancer Research Network of the Fonds de Recherche du Québec - Santé (FRQS), or from AML patient screen samples prior to enrolment in our ribavirin combination therapy clinical trial with Health Canada approval^14^. Blasts were isolated using flow cytometry as described^12^. Specimens from healthy donors were obtained from ATCC and Lonza.

### Plasmids, antibodies and reagents.

pcDNA-2Flag-eIF4E wild-type and vector as well as MSCV-pgk-GFP-eIF4E and control constructs were previously described^18,64,66,72^.

Antibodies for immunoblotting: mouse monoclonal anti-eIF4E (cat# 610270, BD Biosciences), mouse monoclonal anti-β-Actin (cat# A5441, Sigma Aldrich), rabbit polyclonal anti-Mcl-1 (S-19) (cat# sc-819, Santa Cruz), mouse monoclonal anti-HSP90α/β (F-8) (cat# sc-13119, Santa Cruz), rabbit polyclonal anti-Myc (cat# ab32072, Abcam), Mouse monoclonal anti-CD44 antibody (cat# 156–3 C11, Cell Signaling Technology), rabbit polyclonal anti-CD44 (cat# A12410, Abclonal), rabbit polyclonal anti-HAS3 antibody (cat# ab154104, Abcam), rabbit polyclonal anti-phosphoglucomutase 5 (cat# AI14638, Abgent), rabbit polyclonal anti-Lamin A (C-terminal) (cat# L1293, Sigma Aldrich), rabbit polyclonal anti-GAPDH (FL-335) (cat# sc-25778, Santa Cruz), rabbit polyclonal anti-UGDH (cat#AP12613b-EV, Abgent), rabbit monoclonal anti-Hexokinase II (cat# 2867, Cell Signaling Technology), rabbit monoclonal anti-UAP1 antibody (cat# 2716, GenuinBiotech), mouse monoclonal anti-RPL17 (C-8, cat# sc-515904, Santa Cruz), mouse monoclonal anti-RPS16 (D-8, cat# sc-518206; Santa Cruz), mouse monoclonal anti-RPS6 (C-8, cat# sc-74459, Santa Cruz), mouse monoclonal anti-eIF3e (G-7, cat# sc-390413, Santa Cruz), mouse monoclonal anti-eIF3p110 (B-6, cat# sc-74507, Santa Cruz), mouse monoclonal anti-eIF3b (A-7, cat# sc-374156, Santa Cruz), mouse monoclonal anti-eIF4AI/II (H-5, cat# sc-377315, Santa Cruz), mouse monoclonal anti-eIF4G (A-10, cat# sc-133155, Santa Cruz), mouse monoclonal anti-Nopp140 (E-7, cat# sc-374033, Santa Cruz), rabbit monoclonal anti-histone H3 acetyl K27 (cat# ab177178, Abcam), rabbit polyclonal Calreticulin (cat# AW5211, ABCEPTA), rabbit polyclonal anti-Calnexin (cat# ab22595, Abcam), rabbit polyclonal anti-Ezrin antibody (cat# PA5–80603, Invitrogen), mouse monoclonal anti-Ezrin (CPTC-Ezrin-1, cat# AB_2100318, DSHB), mouse monoclonal anti-MEK-1 (H-8, sc-6250, Santa Cruz), and mouse monoclonal anti-cytochrome c (A-8, cat# sc-13156, Santa Cruz). Secondary antibodies: Peroxidase AffiniPure^™^ Goat Anti-Mouse IgG (cat# 115–035-146, Jackson Immunoresearch), Peroxidase AffiniPure^™^ Goat Anti-Rabbit IgG (cat# 111–035-144, Jackson Immunoresearch) and mouse monoclonal anti-puromycin (cat# MABE343, Millipore).

For Immunoprecipitations: rabbit polyclonal anti-Ezrin antibody (cat# PA5–80603, Invitrogen), rabbit polyclonal anti-eIF4E (cat# RN001P, MBL international), mouse monoclonal anti-ribosomal RNA (Y10b, cat# AB_2313703, DSHB), Normal rabbit polyclonal IgG (cat#12–370, Millipore), Normal mouse IgG (cat#12–371, Millipore).

For immunostaining: rat monoclonal anti-hum/mouse CD44 (IM7; cat# 14–0441-82, eBioscience), mouse monoclonal anti-ribosomal RNA (Y10b, cat# AB_2313703, DSHB), rabbit polyclonal anti-Ezrin antibody (cat# PA5–80603, Invitrogen), mouse monoclonal anti-eIF4E (cat#610270, BD Biosciences), rabbit anti-eIF4E (cat# RN001P, MBL international).

Secondary antibodies for immunostaining all from Jackson Immunoresearch: Rhodamine Red-X-conjugated affinity pure Donkey Anti-Mouse IgG (cat# 71529515), Alexa Fluor^®^ 647 AffiniPure Donkey Anti-Rat IgG (H+L) (cat# 712–605-153), Alexa Fluor^®^ 488 AffiniPure Donkey Anti-Mouse IgG (cat# 715–545-151), Alexa Fluor^®^ 488 AffiniPure Goat Anti-Mouse IgG, Fcγ subclass 2a specific (cat# 115–545-206), Brilliant Violet^™^ 480-conjugated AffiniPure Donkey Anti-Rabbit IgG (cat# 711–685-152), Rhodamine Red-X-conjugated affinity pure Donkey Anti-Mouse IgG, subclass 1 specific (cat# 115–295-205), Alexa Fluor^®^ 790 AffiniPure^®^ Donkey Anti-Rat IgG (cat# 712–655-153), Alexa Fluor^®^ 647 AffiniPure^®^ Donkey Anti-Rabbit IgG (cat# 711–605-152).

Ribavirin was purchased from Kemprotek (cat# 36791–04-5), puromycin from Santa Cruz (sc-108071B), Homoharringtonine from MedChemExpress (cat# HY-14944). Hyaluronidase from *Streptomyces Hyalurolyticus* was obtained from Millipore (cat# H1136).

### Western blot analysis

Western blots were performed as described previously^45^, briefly blots were blocked in blocking buffer containing 5% milk in TRIS buffered saline (TBS)–Tween 20. Primary antibodies were diluted in blocking buffer. Quantification of blots was carried out using Fiji (doi:10.1038/nmeth.2019).

### Invasion and adhesion assay

Seventy-two hours prior to the assay, HS-5 cells were seeded into transwell chambers (5×10^4^ cells per 24 well inserts). 24 hours prior to the assay, MM6 cells were seeded in fresh media at near confluent densities (8×10^5^/mL), in 5–10 mL volume. After growing in fresh media for 24 hours, cells were harvested and washed twice with 1xPBS. Cell pellets were resuspended in 10 mL of 5 μM DiIC12(3) perchlorate (cat# ENZ-52206, Enzo Life Sciences) diluted in RPMI medium without additives (1:10 000 from a 50 mM stock solution) and incubated for 10 minutes at 37 °C. After washing three times with 1X PBS, cells were resuspended in 5 mL of complete RPMI medium, counted, diluted to 300 ×10^5^ cells/mL and 0.5 mL of cell suspensions were seeded into the upper chamber of 24-well Transwell fluoroblok inserts (with an 8 μm pore size PET membrane, Cat#351152 Corning) in triplicates, and in parallel the same number of cells were plated into 24 well plates at same density and volume as for upper chamber of flouroblock inserts. Complete growth RPMI medium (0.75 mL) containing 50 ng/mL MCP-1 (279-MC, R&D Systems) was added to the lower chamber. For each experiment plating control wells were seeded, where the same number of cells used for invasion and adhesion assays was seeded in 24 well plates (in technical triplicate for each condition). Thus, we monitored and corrected for errors that could occur in the counting and seeding steps. Cells were incubated for 18–24 hours at 37°C.

For the adhesion assay, HS-5 cells were seeded into 24 well plates 72 hours prior to the assay at 6×10^4^ cells per 24 well. MM6 cells were seeded into a fresh media 24 hours before the experiment as for the invasion assy. After staining with DiIC12(3) perchlorate, cells were seeded into 24 well plates with HS-5 cells at 300 ×10^5^ cells/mL and incubated for 1 hour. Cells were gently washed three times with 1xPBS, culture media was added and plates were used for quantification.

Quantification of invasion and adhesion images of invaded or adhered cells and their plating controls, images were taken using a 2.5x objective fluorescent microscope set to the Cy3 channel (Leica DM IRB Inverted Fluorescence Microscope^®^). The captured images were used for quantification using NIH ImageJ^®^ software (https://imagej.nih.gov/ij). Quantification was also done with Cytation 3 plate reader (BioTek).

### Mesenchymal stromal cell spheroids (3D co-culture system)

Spheroids were established as previously reported^49^. Briefly, HS-5 cells were first seeded at 25,000 into 96-well round-bottom plates pre-coated with 1% UltraPure Agarose (cat# 16500, Invitrogen). Twenty-four hours later, when spheroids were formed, 25,000 AML cells were added per well. AML cell lines, that did not express GFP, were pre-stained with CellTrace^™^ Violet (CTV, cat# C34557, Invitrogen). At 24 hours of co-culture, the spheroid was picked up using a 200 μL-pipette tip, washed with DPBS-5mM EDTA (cat# 15575–038, Invitrogen), and enzymatically disaggregated with 0.25% Trypsin/2.21 mM EDTA (cat# 25–053, Corning) for 10 minutes, followed by mechanical disruption. Single cell suspensions were stained with 7-AminoActinomycin D (7-AAD, cat# A1310, Invitrogen) as viability dye and APC Cy7 anti-human CD45 (hCD45) antibody (cat# 368516, BioLegend).

### AML mouse models

All animal procedures were conducted under protocols approved by the Weill Cornell Medicine Institutional Animal Care and Use Committee (WCM-IACUC). Female, 8–10-week-old, non-obese diabetic/severe combined immunodeficient IL2Rgamma^null^ (NSG) mice were obtained from the Jackson Laboratories (strain #:005557).

For cell line derived xenografts (CDX), MM6 CRISPR cell lines CTRL or 4E clones (5.5 × 10^5^), and THP-1 CRISPR cell lines CTRL or 4E clones (4 × 10^6^) were injected via tail vein into sublethally irradiated NSG mice (200 rad). Bone marrow (BM) and spleens were harvested to assess leukemia cell engraftment. Cells were stained with PE-Cy5 anti-mouse CD45 (mCD45) (cat# 103110, BioLegend) and APC Cy7 anti-hCD45 (cat# 368516, BioLegend) antibodies to distinguish human cells from host mouse cells. 4’,6-diamidino-2-phenylindole (DAPI, cat# D1306, Invitrogen) or fixable viability dye eFluor^™^ 450 (cat# 65–0863-14, Invitrogen) were added to assess viability. Additional monitoring was done using BM aspirates and overall survival was also assessed. Mice exhibiting signs of distress were euthanized in accordance with WCM-IACUC guidelines. We confirmed by western blot that MM6 CRISPR 4E and THP-1 CRISPR 4E cells remained deficient in eIF4E at the endpoint, briefly AML cells (hCD45^+^) were column-purified from mouse BM samples using anti-human CD45 MicroBeads (cat# 130–045-801, Miltenyi Biotec).

AML patient derived xenografts (PDX) models were established based on previous studies^73,74^. Briefly, primary AML cells (2.5 × 10^6^ cells per mouse) were injected into sublethally irradiated NSG mice (200 rad). Seventeen days post-cell injections, mice were treated via intraperitoneal injection with either 100 mg/kg of Ribavirin (cat# S2504, Selleck Chemicals) or vehicle control (saline) daily for 28 days, followed by a 44-day drug-free period. Treatment cycle was then repeated using 100 mg/kg for 20 days. Peripheral blood was periodically monitored to assess leukemia burden, as described previously^74^. Overall survival was also evaluated.

### Immunofluorescence and laser-scanning confocal microscopy

MM6 cells grown in liquid cultures (suspension) or collected from the bottom chamber of transwell system from the invasion assays (invaded; as shown in Supplemental Figure 6A) were fixed with 3% paraformaldehyde (PFA, cat# 158127, SIGMA) for 15 minutes at room temperature (RT) with gentle shaking (PFA was added directly to the growth media), followed by quenching with 150mM glycine (BP381–5, Fisher)for 5 minutes at RT with gentle shaking (glycine was added directly to cells in media with PFA). Cells were harvested and washed three times in 1 x PBS in eppendorf tubes, using centrifugation, 5 minutes at 1200 rpm. Washed pellets were resuspended in 1 x PBS at 0.5–1×10^5^ cells/10μl, and 10μl cell suspensions were spotted on glass slides. After drying, slides were washed once with 1 x PBS, permeabilized with 0.5% Triton X-100 (T-9284, Sigma) for 10 minutes at RT, washed three times with 1 x PBS, blocked for 1 h in blocking solution (10% FBS and 0.1% Tween 20 in PBS) and incubated with primary antibodies diluted in blocking solution overnight at 4°C. After washing, cells were incubated with fluorescently labelled secondary antibodies diluted in blocking solution for 1h at RT, washed three times in 1 x PBS and mounted in ProLong^™^ gold antifade media with DAPI (cat# P36935, Invitrogen).

Imaging was carried out using the Zeiss LSM700 with 63x oil objective and numerical aperture of 1.4 (Figures 1A and 1B, Supplemental Figure 1E); Leica TCS SP8 DLS line scan confocal microscope, with 63x oil objective and numerical aperture of 1.4 (Figure 3J, Supplemental Figure 1B, with z-stack step 0.3 μm); the Nikon AXR confocal microscope with 60x oil objective and numerical aperture of 1.42 (Figures 4D and 5B, Supplemental Figure 6B, with z-stack step 0.2 μm); or the Nikon CSU-W1 Dual CAM spinning disc microscope with 60x oil objective and numerical aperture 1.42 (Figures 4E, 4F, 5C, with z-stack step 0.3 μm) or 100x oil objective and numerical aperture 1.45 (Figure 4B, with z-stack step 0.3 μm). Channels were detected separately, with no crosstalk observed. Confocal micrographs represent single sections through the plane of the cell. Confocal micrographs represent single sections through the plane of the cell. Images were obtained from ZEN (Carl Zeiss, Inc.) or FIJI software.

### Visualizing Translation Activity using RiboLace (VISTA-R) staining

MM6 cells were fixed and permeabilized as described in the previous section (see Supplemental Figure 6A). After washing with 1 x PBS, cells were then washed twice with RL buffer (20mM HEPES pH=7.4, 10mM MgCl_2_, 137mM NaCl, 2.7mM KCl) and blocked in 2% bovine serum albumin (BSA, cat# BP9703–100, Fisher Scientific) with 50mg/ml streptavidin (cat# 434302, Invitrogen) in RL buffer for 30 minutes at RT. Cells were then washed three times with RL buffer and blocked for 30 minutes at RT with 2% BSA with 2mg/ml Biotin (cat# B1595, Invitrogen) in RL buffer. After washing three times with RL buffer, cells were incubated for 1 hour at RT with 50mM Biotinylated Puromycin (cat# 2379889–82-2, MedChemExpress) in RL buffer with 2% BSA. After washing three times with RL buffer, cells were incubated with 2mg/ml NeutrAvidin Oregon Green 488 (cat# A6374, Invitrogen) in 2% BSA in RL buffer for 1 hour at RT, washed three times with RL buffer and either mounted in ProLong^™^ gold antifade media with DAPI, or stained for proteins as indicated in the text. In the case of immunostaining after VISTA-R, cells were blocked for 1 hour in blocking solution (10% FBS and 0.1% Tween 20 in PBS) and incubated with primary antibodies diluted in blocking solution overnight at 4°C. After washing, cells were incubated with fluorescently labelled secondary antibodies (all from Jackson Immunoresearch) diluted in blocking solution for 1 hour at RT, washed three times in 1xPBS and mounted in ProLong^™^ gold antifade media with DAPI.

### Imaris-Based Quantification of T-PODs

All image processing and quantitative analyses were performed using Imaris v10.2 (Oxford Instruments). The Distance Transformation XTension (MATLAB) was used for nuclear (DAPI) surface expansion. DAPI surfaces were generated using automatic absolute intensity thresholding without the use of creation parameters and filtering. The distance transformation XTension was applied in Outside SurfaceObject mode to compute a distance channel from nuclear boundaries. A distance surface was generated using threshold bounds of 0.00–1.70, capturing cytoplasmic regions from the nuclear envelope to the plasma membrane. Ezrin, VISTA-R, and rRNA (Y10b) surfaces were generated using background subtraction (Local Contrast) with a largest sphere diameter of 10 μm and automatic Absolute intensity thresholding. Object-Object statistics was disabled. Surfaces were filtered to remove background, multicellular aggregates, and non-viable cells. Filtering parameters differed slightly between experimental conditions (CTRL 7 vs. CTRL 13) based on different staining days but followed consistent biological criteria: retention of single, viable cells completely in view of the image file. For CTRL 7, Ezrin surfaces were filtered using a voxel number threshold of > 8,000, area < 950 μm^2^, mean intensity (CD44 channel) > 100, and minimum intensity (Ezrin channel) > 100. VISTA-R and rRNA surfaces were filtered more stringently to account for additional background (voxel number > 1.5×10^4^ for VISTA-R and > 2.7×10^4^ for rRNA; area 150–950 μm^2^), with additional spatial constraints excluding objects within 3–5 μm of image borders in the X and Y dimensions, if necessary. To exclude DAPI-positive non-viable cells, which exhibited abnormal nuclear signals, summed and maximum intensity thresholds were applied to the relevant DAPI channels (Intensity Sum > 1.47×10^7^ and Intensity Max > 1.00×10^−5^). These thresholds were empirically defined from the distribution of nuclear intensities across the dataset and removed cells including atypical nuclear signals. For CTRL 13, Ezrin, VISTA-R and rRNA surfaces were filtered using a minimum voxel threshold of > 8,000, area < 950 μm^2^, mean intensity (CD44 channel) > 100, and minimum intensity (Ezrin channel) > 100, without additional spatial or viability-based intensity constraints, as they were unnecessary.

To isolate T-POD associated signal, Ezrin, VISTA-R and rRNA channels were masked using the distance surface by setting voxel intensities inside to 0.00, producing nucleus- and perinuclear-excluded (T-POD) channels (Supplemental Figure 6D). Final surfaces were refined by excluding zero-intensity objects (mean intensity > 0.00001) corresponding to the newly generated T-POD channels. Surface-based measurements were extracted using the Vantage module. Quantification was performed on Ezrin, VISTA-R, and rRNA surfaces using their corresponding T-POD channels. Identical segmentation and filtering parameters were applied across all image files within the same experimental condition and acquisition time. Intensities per T-POD per channel was calculated using intensity Sum.

### Reverse transcription and quantitative PCR

RNA samples were reversed transcribed using SuperScript VILO (cat# 11754–050, Invitrogen) and Superscript IV cDNA (cat# 11756050, Invitrogen) synthesis kits (for RIP experiments) or MMLV reverse transcription kit using oligo-dT or Random hexamers (cat# 28025013, Invitrogen) for analysis of total RNA levels. qPCR analyses were performed using PowerUp Sybr Green Master Mix (cat# A25777, Applied Biosystems) or Sybr Green qPCR Master Mix (cat# GK10002, GLPBio) in Applied Biosystems Viia7, QuantStudio7 or QuantStudio7 thermal cyclers using the relative standard curve method (Applied Biosystems User Bulletin #2). All the primers are listed in Supplemental Table 1. Cycling conditions 5 minutes at 95°C followed by 40 cycles of denaturation at 95°C for 5 sec and annealing and extension at 60°C for 30 seconds except for Hk2 and HAS3 primers which were carried at 62°C for invaded cells which had low RNA content.

### Crosslinking and fractionation of cells for IPs and RIPs

For IPs from cross-linked cells, 1–2×10^7^ cells in suspension were fixed with 1% PFA final concentration (directly added to culture media) and incubated with slow shaking 15 min at RT. PFA was quenched with 0.15 M Glycine (final concentration, directly added to cell suspension) and incubated 5 min at RT with slow shaking. For total cell lysates, after washing three times with 1 x PBS, cell pellets were resuspended in 1ml NT-2 buffer and divided into two eppendorf tubes (1.5 ml) and sonicated three times with 5 seconds bursts (30 second pause between each burst) using microtip at 25% power (Sonic Dismembrator Model 500, Fisher, Max Output 400W). For fractionation of crosslinked cells, ~3×10^6^ fixed cells were resuspended in 0.5 ml NT2 buffer and sonicated with three rounds of 5 seconds bursts/30 seconds pause with microtip at power 12. Lysates were centrifuged 3 minutes at 1000×g, cytosolic fraction was transferred into fresh tube, and pellets (nuclei) were pulled and resuspended in 100 ml of NT-2 buffer and sonicated three times 5 seconds burst/30 seconds pause with microtip at power 20. Nuclear and cytoplasmic lysates were centrifuged for 10 minutes at 10 000×g and supernatants were transferred into fresh tubes. NT-2 buffer: 150 mM NaCl, 50 mM Tris-HCl (pH 7.4), 2.5 mM MgCl_2_, 0.05% Nonidet P-40, supplemented with 1mM DTT, 2x protease inhibitors without EDTA, 200U/ml RNaseOut. Nuclear lysates were centrifuged at 10 000 × g for 10 minutes, and supernatants were transferred into fresh tubes.

### Co-Immunoprecipitation and RIP

Total cell lysates or cytoplasmic fractions (from cells fixed with PFA) adjusted to be no more than 1 mg/ml concentration, were pre-cleared with 50 μL protein G conjugated superparamagnetic beads (Dynabeads Protein G, ThermoFisher Scientific) for 30 min at 4°C. Pre-cleared lysates (1mg total cell lysates or 100–500mg cytoplasmic lysates) were incubated with 10μg of anti-eIF4E antibody (RN001P, MBL), 8 μg of anti-Ezrin antibody (cat# PA5–80603, Invitrogen), or 10 μg of rabbit IgG (cat# 12–370, Millipore) as a control, and 0.5 mg/ml yeast tRNA (cat# R8508, SigmaAldrich), overnight at 4°C with rotation. After ON incubation, 50 μl of Dynabeads (cat# 1004D, Invitrogen) were added and incubated for additional 3h at 4°C with rotation. Beads were washed once with NT-2 buffer Supplemented with 1mg/mL heparin (cat# 353003, Sigma-Aldrich) for 5 minutes at 4°C with rotation, and an additional six times with NT-2 buffer with 300 mM NaCl. In case of Y10b IPs, 100 ml of Y10b mouse hybridoma supernatant (DSHB), or 10 mg od mouse IgG (cat# 12–371, Millipore) were bound to the beads 50 μL protein G conjugated superparamagnetic beads in 1 ml of NT2 buffer ON at 4°C, washed three times with NT2 buffer, added to 1 mg of total cell lysates, incubated overnight at 4°C with rotation and washed as described for IPs with rabbit antibodies. After washing, beads were resuspended in 2 x Laemmli buffer (cat# 1610737, Bio-Rad) with β-mercaptoethanol and incubated for 5 minutes at 98°C. Co-immunoprecipitated proteins were resolved on SDS-PAGE and visualized by western blotting. To isolate RNAs from immunoprecipitated reactions, beads were resuspended in elution buffer (100 mM Tris-HCl (pH 6.8), 4% (w/v) SDS, 20% (v/v) glycerol, 12% (v/v) β-mercaptoethanol (444203, Sigma), and incubated for 5 minutes at 98°C. RNA were isolated using TRIzol reagent (cat# 15596018, Invitrogen) and Direct-zol RNA Micro-prep Kit (cat# R2050,Zymo Research).

### Statistical analyses

Graphs and statistics were generated using GraphPad Prism Version 10.6.1. Biological replicates and p-values are provided in each figure and the statistical test applied is named in the legend.

## Supplementary Material

Supplement 1

## Highlights:

- eIF4E reduction impairs AML cell motility and disease progression
- eIF4E-dependent motility requires Ezrin
- Ezrin binds eIF4E, transcripts encoding motility factors and active ribosomes
- VISTA-R enabled visualization of active ribosomes and translationally active pseudopods (T-PODs)
- T-PODs provide novel on-demand localized translation to sustain mobility at migratory sites

## References

[1] Wysota M., Konopleva M., and Mitchell S. (2024). Novel Therapeutic Targets in Acute Myeloid Leukemia (AML). Curr Oncol Rep 26, 409–420. 10.1007/s11912-024-01503-y. 38502417 PMC11021231

[2] Hayman T.J., Williams E.S., Jamal M., Shankavaram U.T., Camphausen K., and Tofilon P.J. (2012). Translation initiation factor eIF4E is a target for tumor cell radiosensitization. Cancer Res 72, 2362–2372. 10.1158/0008-5472.CAN-12-0329. 22397984 PMC6327305

[3] Wang A., and Zhong H. (2018). Roles of the bone marrow niche in hematopoiesis, leukemogenesis, and chemotherapy resistance in acute myeloid leukemia. Hematology 23, 729–739. 10.1080/10245332.2018.1486064. 29902132

[4] Borella G., Da Ros A., Borile G., Porcu E., Tregnago C., Benetton M., Marchetti A., Bisio V., Montini B., Michielotto B., (2021). Targeting the plasticity of mesenchymal stromal cells to reroute the course of acute myeloid leukemia. Blood 138, 557–570. 10.1182/blood.2020009845. 34010415

[5] Behrmann L., Wellbrock J., and Fiedler W. (2018). Acute Myeloid Leukemia and the Bone Marrow Niche-Take a Closer Look. Front Oncol 8, 444. 10.3389/fonc.2018.00444. 30370251 PMC6195156

[6] Ganzel C., Manola J., Douer D., Rowe J.M., Fernandez H.F., Paietta E.M., Litzow M.R., Lee J.W., Luger S.M., Lazarus H.M., (2016). Extramedullary Disease in Adult Acute Myeloid Leukemia Is Common but Lacks Independent Significance: Analysis of Patients in ECOG-ACRIN Cancer Research Group Trials, 1980–2008. J Clin Oncol 34, 3544–3553. 10.1200/JCO.2016.67.5892. 27573652 PMC5074349

[7] Ohanian M., Faderl S., Ravandi F., Pemmaraju N., Garcia-Manero G., Cortes J., and Estrov Z. (2013). Is acute myeloid leukemia a liquid tumor? Int J Cancer 133, 534–543. 10.1002/ijc.28012. 23280377 PMC3904286

[8] Mars J.C., Culjkovic-Kraljacic B., and Borden K.L.B. (2024). eIF4E orchestrates mRNA processing, RNA export and translation to modify specific protein production. Nucleus 15, 2360196. 10.1080/19491034.2024.2360196. 38880976 PMC11185188

[9] Ghram M., Morris G., Culjkovic-Kraljacic B., Mars J.C., Gendron P., Skrabanek L., Revuelta M.V., Cerchietti L., Guzman M.L., and Borden K.L.B. (2023). The eukaryotic translation initiation factor eIF4E reprograms alternative splicing. EMBO J 42, e110496. 10.15252/embj.2021110496. 36843541 PMC10068332

[10] Graff J.R., and Zimmer S.G. (2003). Translational control and metastatic progression: enhanced activity of the mRNA cap-binding protein eIF-4E selectively enhances translation of metastasis-related mRNAs. Clin Exp Metastasis 20, 265–273. 10.1023/a:1022943419011. 12741684

[11] Borden K.L., and Culjkovic-Kraljacic B. (2010). Ribavirin as an anti-cancer therapy: acute myeloid leukemia and beyond? Leuk Lymphoma 51, 1805–1815. 10.3109/10428194.2010.496506. 20629523 PMC2950216

[12] Assouline S., Culjkovic B., Cocolakis E., Rousseau C., Beslu N., Amri A., Caplan S., Leber B., Roy D.C., Miller W.H. Jr., and Borden K.L. (2009). Molecular targeting of the oncogene eIF4E in acute myeloid leukemia (AML): a proof-of-principle clinical trial with ribavirin. Blood 114, 257–260. 10.1182/blood-2009-02-205153. 19433856

[13] Assouline S., Culjkovic-Kraljacic B., Bergeron J., Caplan S., Cocolakis E., Lambert C., Lau C.J., Zahreddine H.A., Miller W.H. Jr., and Borden K.L. (2015). A phase I trial of ribavirin and low-dose cytarabine for the treatment of relapsed and refractory acute myeloid leukemia with elevated eIF4E. Haematologica 100, e7–9. 10.3324/haematol.2014.111245. 25425688 PMC4281321

[14] Assouline S., Gasiorek J., Bergeron J., Lambert C., Culjkovic-Kraljacic B., Cocolakis E., Zakaria C., Szlachtycz D., Yee K., and Borden K.L.B. (2023). Molecular targeting of the UDP-glucuronosyltransferase enzymes in high-eukaryotic translation initiation factor 4E refractory/relapsed acute myeloid leukemia patients: a randomized phase II trial of vismodegib, ribavirin with or without decitabine. Haematologica. 10.3324/haematol.2023.282791. PMC10620574 36951168

[15] Zhou F., Culjkovic-Kraljacic B., Bach C., Feng L., Mishima Y., Borden K.L.B., and Tenen D.G. (2025). Posttranscriptional activity of eIF4E contributes to HoxA9-driven leukemogenesis. Blood Neoplasia 2, 100132. 10.1016/j.bneo.2025.100132. 40979070 PMC12447889

[16] Topisirovic I., and Borden K.L. (2005). Homeodomain proteins and eukaryotic translation initiation factor 4E (eIF4E): an unexpected relationship. Histol Histopathol 20, 1275–1284. 10.14670/HH-20.1275. 16136508

[17] Topisirovic I., Culjkovic B., Neering S.J., Jordan C.T., and Borden K.L. (2005). HoxA9 is a stimulator of eIF4E dependent mRNA transport. Mol Cell Biol 25, 1100–1112.15657436

[18] Topisirovic I., Guzman M.L., McConnell M.J., Licht J.D., Culjkovic B., Neering S.J., Jordan C.T., and Borden K.L. (2003). Aberrant eukaryotic translation initiation factor 4E-dependent mRNA transport impedes hematopoietic differentiation and contributes to leukemogenesis. Mol Cell Biol 23, 8992–9002. 10.1128/MCB.23.24.8992-9002.2003. 14645512 PMC309660

[19] Kraljacic B.C., Arguello M., Amri A., Cormack G., and Borden K. (2011). Inhibition of eIF4E with ribavirin cooperates with common chemotherapies in primary acute myeloid leukemia specimens. Leukemia 25, 1197–1200. 10.1038/leu.2011.57. 21455212

[20] Zahreddine H.A., Culjkovic-Kraljacic B., Assouline S., Gendron P., Romeo A.A., Morris S.J., Cormack G., Jaquith J.B., Cerchietti L., Cocolakis E., (2014). The sonic hedgehog factor GLI1 imparts drug resistance through inducible glucuronidation. Nature 511, 90–93. 10.1038/nature13283. 24870236 PMC4138053

[21] Kentsis A., Topisirovic I., Culjkovic B., Shao L., and Borden K.L. (2004). Ribavirin suppresses eIF4E-mediated oncogenic transformation by physical mimicry of the 7-methyl guanosine mRNA cap. Proc Natl Acad Sci U S A 101, 18105–18110. 10.1073/pnas.0406927102. 15601771 PMC539790

[22] Volpon L., Osborne M.J., Zahreddine H., Romeo A.A., and Borden K.L. (2013). Conformational changes induced in the eukaryotic translation initiation factor eIF4E by a clinically relevant inhibitor, ribavirin triphosphate. Biochemical and biophysical research communications 434, 614–619. 10.1016/j.bbrc.2013.03.125. 23583375 PMC3659399

[23] Kentsis A., Volpon L., Topisirovic I., Soll C.E., Culjkovic B., Shao L., and Borden K.L. (2005). Further evidence that ribavirin interacts with eIF4E. RNA 11, 1762–1766. 10.1261/rna.2238705. 16251386 PMC1370864

[24] Bollmann F., Fechir K., Nowag S., Koch K., Art J., Kleinert H., and Pautz A. (2013). Human inducible nitric oxide synthase (iNOS) expression depends on chromosome region maintenance 1 (CRM1)- and eukaryotic translation initiation factor 4E (elF4E)-mediated nucleocytoplasmic mRNA transport. Nitric Oxide 30, 49–59. 10.1016/j.niox.2013.02.083. 23471078

[25] Volpin F., Casaos J., Sesen J., Mangraviti A., Choi J., Gorelick N., Frikeche J., Lott T., Felder R., Scotland S.J., (2017). Use of an anti-viral drug, Ribavirin, as an anti-glioblastoma therapeutic. Oncogene 36, 3037–3047. 10.1038/onc.2016.457. 27941882

[26] Shi F., Len Y., Gong Y., Shi R., Yang X., Naren D., and Yan T. (2015). Ribavirin Inhibits the Activity of mTOR/eIF4E, ERK/Mnk1/eIF4E Signaling Pathway and Synergizes with Tyrosine Kinase Inhibitor Imatinib to Impair Bcr-Abl Mediated Proliferation and Apoptosis in Ph+ Leukemia. PLoS One 10, e0136746. 10.1371/journal.pone.0136746. 26317515 PMC4552648

[27] Jin J., Xiang W., Wu S., Wang M., Xiao M., and Deng A. (2019). Targeting eIF4E signaling with ribavirin as a sensitizing strategy for ovarian cancer. Biochem Biophys Res Commun 510, 580–586. 10.1016/j.bbrc.2019.01.117. 30739792

[28] Dai D., Chen H., Tang J., and Tang Y. (2017). Inhibition of mTOR/eIF4E by anti-viral drug ribavirin effectively enhances the effects of paclitaxel in oral tongue squamous cell carcinoma. Biochem Biophys Res Commun 482, 1259–1264. 10.1016/j.bbrc.2016.12.025. 27932243

[29] Pettersson F., Del Rincon S.V., Emond A., Huor B., Ngan E., Ng J., Dobocan M.C., Siegel P.M., and Miller W.H. Jr. (2015). Genetic and pharmacologic inhibition of eIF4E reduces breast cancer cell migration, invasion, and metastasis. Cancer Res 75, 1102–1112. 10.1158/0008-5472.CAN-14-1996. 25608710

[30] Dunn L.A., Fury M.G., Sherman E.J., Ho A.A., Katabi N., Haque S.S., and Pfister D.G. (2018). Phase I study of induction chemotherapy with afatinib, ribavirin, and weekly carboplatin and paclitaxel for stage IVA/IVB human papillomavirus-associated oropharyngeal squamous cell cancer. Head Neck 40, 233–241. 10.1002/hed.24938. 28963790 PMC6760238

[31] Kosaka T., Maeda T, Shinojima T, Nagata H, Ryuchi R, Oya M (2017). a clinical study to evaluate the efficacy and safety of docetaxal with ribavirin in patients with progressive castration resistant prostate cancer who have previously received docetaxol alone. Journal of Clinical Oncology, https://ascopubs.org/doi/abs/10.1200/JCO2017.1235.1215_suppl.e14010.

[32] Burman B., Drutman S.B., Fury M.G., Wong R.J., Katabi N., Ho A.L., and Pfister D.G. (2022). Pharmacodynamic and therapeutic pilot studies of single-agent ribavirin in patients with human papillomavirus-related malignancies. Oral Oncol 128, 105806. 10.1016/j.oraloncology.2022.105806. 35339025 PMC9788648

[33] Osborne M.J., Coutinho de Oliveira L., Volpon L., Zahreddine H.A., and Borden K.L.B. (2019). Overcoming Drug Resistance through the Development of Selective Inhibitors of UDP-Glucuronosyltransferase Enzymes. J Mol Biol 431, 258–272. 10.1016/j.jmb.2018.11.007. 30428301 PMC6331242

[34] Zahreddine H.A., Culjkovic-Kraljacic B., Gasiorek J., Duchaine J., and Borden K.L.B. (2019). GLI1-Inducible Glucuronidation Targets a Broad Spectrum of Drugs. ACS Chem Biol 14, 348–355. 10.1021/acschembio.8b01118. 30763062

[35] Osborne M.J., Sulekha A., Culjkovic-Kraljacic B., Gasiorek J., Ruediger E., Jolicouer E., Marinier A., Assouline S., and Borden K.L.B. (2024). Medicinal Chemistry and NMR Driven Discovery of Novel UDP-glucuronosyltransferase 1A Inhibitors That Overcome Therapeutic Resistance in Cells. J Mol Biol 436, 168378. 10.1016/j.jmb.2023.168378. 38043731 PMC10841659

[36] Martinez L.M., and Guzman M.L. (2024). Understanding the interaction between leukaemia stem cells and their microenvironment to improve therapeutic approaches. Br J Pharmacol 181, 273–282. 10.1111/bph.16162. 37309573 PMC13343339

[37] Chang F., Kong S.J., Wang L., Choi B.K., Lee H., Kim C., Kim J.M., and Park K. (2020). Targeting Actomyosin Contractility Suppresses Malignant Phenotypes of Acute Myeloid Leukemia Cells. Int J Mol Sci 21. 10.3390/ijms21103460. PMC7279019 32422910

[38] George S., Martin J.A.J., Graziani V., and Sanz-Moreno V. (2022). Amoeboid migration in health and disease: Immune responses versus cancer dissemination. Front Cell Dev Biol 10, 1091801. 10.3389/fcell.2022.1091801. 36699013 PMC9869768

[39] Gutjahr J.C., Bayer E., Yu X., Laufer J.M., Hopner J.P., Tesanovic S., Harzschel A., Auer G., Riess T., Salmhofer A., (2021). CD44 engagement enhances acute myeloid leukemia cell adhesion to the bone marrow microenvironment by increasing VLA-4 avidity. Haematologica 106, 2102–2113. 10.3324/haematol.2019.231944. 32616529 PMC8327716

[40] Zoller M. (2015). CD44, Hyaluronan, the Hematopoietic Stem Cell, and Leukemia-Initiating Cells. Front Immunol 6, 235. 10.3389/fimmu.2015.00235. 26074915 PMC4443741

[41] Misra S., Hascall V.C., Markwald R.R., and Ghatak S. (2015). Interactions between Hyaluronan and Its Receptors (CD44, RHAMM) Regulate the Activities of Inflammation and Cancer. Front Immunol 6, 201. 10.3389/fimmu.2015.00201. 25999946 PMC4422082

[42] Qu C., Rilla K., Tammi R., Tammi M., Kroger H., and Lammi M.J. (2014). Extensive CD44-dependent hyaluronan coats on human bone marrow-derived mesenchymal stem cells produced by hyaluronan synthases HAS1, HAS2 and HAS3. Int J Biochem Cell Biol 48, 45–54. 10.1016/j.biocel.2013.12.016. 24406795

[43] Arpin M., Chirivino D., Naba A., and Zwaenepoel I. (2011). Emerging role for ERM proteins in cell adhesion and migration. Cell Adh Migr 5, 199–206. 10.4161/cam.5.2.15081. 21343695 PMC3084986

[44] Song X., Wang W., Wang H., Yuan X., Yang F., Zhao L., Mullen M., Du S., Zohbi N., Muthusamy S., (2020). Acetylation of ezrin regulates membrane-cytoskeleton interaction underlying CCL18-elicited cell migration. J Mol Cell Biol 12, 424–437. 10.1093/jmcb/mjz099. 31638145 PMC7333480

[45] Zahreddine H.A., Culjkovic-Kraljacic B., Emond A., Pettersson F., Midura R., Lauer M., Del Rincon S., Cali V., Assouline S., Miller W.H., (2017). The eukaryotic translation initiation factor eIF4E harnesses hyaluronan production to drive its malignant activity. Elife 6. 10.7554/eLife.29830. PMC5705209 29111978

[46] Ghram M., Morris G, Culjkovic-Kraljacic B, Mars JC, Gendron P, Skrabanek L., Revuelta MV, Cerchietti L, Guzman ML, Borden KLB. (2023). Th eukaryotic translation initiation factor eIF4E reprogrammes the splicing machineryand drivesalternative splicing. EMBOJ Apr 3;42(7) :e110496. 10.15252/embj.2021110496. PMC10068332 36843541

[47] Culjkovic-Kraljacic B., Skrabanek L.A., Revuelta M.V., Gasiorek J., Cowling V.H., Cerchietti L., Borden KLB (2020). The eukaryotic translation initiation factor eIF4E elevates m7G capping efficiency of selected coding and non-coding transcripts PNAS Oct 27;117(43): 26773–26783.doi: 10.1073/pnas.2002360117. 33055213 PMC7604501

[48] Adamo A., Delfino P., Gatti A., Bonato A., Takam Kamga P., Bazzoni R., Ugel S., Mercuri A., Caligola S., and Krampera M. (2020). HS-5 and HS-27A Stromal Cell Lines to Study Bone Marrow Mesenchymal Stromal Cell-Mediated Support to Cancer Development. Front Cell Dev Biol 8, 584232. 10.3389/fcell.2020.584232. 33251214 PMC7674674

[49] Balandran J.C., Davila-Velderrain J., Sandoval-Cabrera A., Zamora-Herrera G., Teran-Cerqueda V., Garcia-Stivalet L.A., Limon-Flores J.A., Armenta-Castro E., Rodriguez-Martinez A., Leon-Chavez B.A., (2021). Patient-Derived Bone Marrow Spheroids Reveal Leukemia-Initiating Cells Supported by Mesenchymal Hypoxic Niches in Pediatric B-ALL. Front Immunol 12, 746492. 10.3389/fimmu.2021.746492. 34737747 PMC8561951

[50] Lerner E.A., Lerner M.R., Janeway C.A. Jr., and Steitz J.A. (1981). Monoclonal antibodies to nucleic acid-containing cellular constituents: probes for molecular biology and autoimmune disease. Proc Natl Acad Sci U S A 78, 2737–2741. 10.1073/pnas.78.5.2737. 6789322 PMC319432

[51] Lejbkowicz F., Goyer C., Darveau A., Neron S., Lemieux R., and Sonenberg N. (1992). A fraction of the mRNA 5’ cap-binding protein, eukaryotic initiation factor 4E, localizes to the nucleus. Proc Natl Acad Sci U S A 89, 9612–9616. 10.1073/pnas.89.20.9612. 1384058 PMC50182

[52] Dostie J., Lejbkowicz F., and Sonenberg N. (2000). Nuclear eukaryotic initiation factor 4E (eIF4E) colocalizes with splicing factors in speckles. J Cell Biol 148, 239–247. 10.1083/jcb.148.2.239. 10648556 PMC2174286

[53] Cohen N., Sharma M., Kentsis A., Perez J.M., Strudwick S., and Borden K.L. (2001). PML RING suppresses oncogenic transformation by reducing the affinity of eIF4E for mRNA. EMBO J 20, 4547–4559. 10.1093/emboj/20.16.4547. 11500381 PMC125576

[54] Iborra F.J., Jackson D.A., and Cook P.R. (2001). Coupled transcription and translation within nuclei of mammalian cells. Science 293, 1139–1142. 10.1126/science.1061216. 11423616

[55] Enam S.U., Zinshteyn B., Goldman D.H., Cassani M., Livingston N.M., Seydoux G., and Green R. (2020). Puromycin reactivity does not accurately localize translation at the subcellular level. Elife 9. 10.7554/eLife.60303. PMC7490009 32844748

[56] Clamer M., Tebaldi T., Lauria F., Bernabo P., Gomez-Biagi R.F., Marchioretto M., Kandala D.T., Minati L., Perenthaler E., Gubert D., (2018). Active Ribosome Profiling with RiboLace. Cell Rep 25, 1097–1108 e1095. 10.1016/j.celrep.2018.09.084. 30355487

[57] Hobson B.D., Kong L., Hartwick E.W., Gonzalez R.L., and Sims P.A. (2020). Elongation inhibitors do not prevent the release of puromycylated nascent polypeptide chains from ribosomes. Elife 9. 10.7554/eLife.60048. PMC7490010 32844746

[58] Graff J.R., Konicek B.W., Vincent T.M., Lynch R.L., Monteith D., Weir S.N., Schwier P., Capen A., Goode R.L., Dowless M.S., (2007). Therapeutic suppression of translation initiation factor eIF4E expression reduces tumor growth without toxicity. J Clin Invest 117, 2638–2648. 10.1172/JCI32044. 17786246 PMC1957541

[59] Mori T., Kitano K., Terawaki S., Maesaki R., Fukami Y., and Hakoshima T. (2008). Structural basis for CD44 recognition by ERM proteins. J Biol Chem 283, 29602–29612. 10.1074/jbc.M803606200. 18753140 PMC2662033

[60] Hingorani S.R., Harris W.P., Beck J.T., Berdov B.A., Wagner S.A., Pshevlotsky E.M., Tjulandin S.A., Gladkov O.A., Holcombe R.F., Korn R., (2016). Phase Ib Study of PEGylated Recombinant Human Hyaluronidase and Gemcitabine in Patients with Advanced Pancreatic Cancer. Clin Cancer Res 22, 2848–2854. 10.1158/1078-0432.CCR-15-2010. 26813359 PMC7787348

[61] Hingorani S.R., Zheng L., Bullock A.J., Seery T.E., Harris W.P., Sigal D.S., Braiteh F., Ritch P.S., Zalupski M.M., Bahary N., (2018). HALO 202: Randomized Phase II Study of PEGPH20 Plus Nab-Paclitaxel/Gemcitabine Versus Nab-Paclitaxel/Gemcitabine in Patients With Untreated, Metastatic Pancreatic Ductal Adenocarcinoma. J Clin Oncol 36, 359–366. 10.1200/JCO.2017.74.9564. 29232172

[62] Menke-van der Houven van Oordt C.W., Gomez-Roca C., van Herpen C., Coveler A.L., Mahalingam D., Verheul H.M., van der Graaf W.T., Christen R., Ruttinger D., Weigand S., (2016). First-in-human phase I clinical trial of RG7356, an anti-CD44 humanized antibody, in patients with advanced, CD44-expressing solid tumors. Oncotarget 7, 80046–80058. 10.18632/oncotarget.11098. 27507056 PMC5346770

[63] Chen C., Zhao S., Karnad A., and Freeman J.W. (2018). The biology and role of CD44 in cancer progression: therapeutic implications. J Hematol Oncol 11, 64. 10.1186/s13045-018-0605-5. 29747682 PMC5946470

[64] Culjkovic-Kraljacic B., Baguet A., Volpon L., Amri A., and Borden K.L.B. (2012). The oncogene eIF4E reprograms the nuclear pore complext to promote mRNA export and oncogenic transformation. Cell Reports 2, 207–215. 22902403 10.1016/j.celrep.2012.07.007 PMC3463940

[65] Culjkovic B., Topisirovic I., Skrabanek L., Ruiz-Gutierrez M., and Borden K.L. (2005). eIF4E promotes nuclear export of cyclin D1 mRNAs via an element in the 3’UTR. J Cell Biol 169, 245–256. 10.1083/jcb.200501019. 15837800 PMC2171863

[66] Culjkovic B., Topisirovic I., Skrabanek L., Ruiz-Gutierrez M., and Borden K.L. (2006). eIF4E is a central node of an RNA regulon that governs cellular proliferation. J Cell Biol 175, 415–426. 10.1083/jcb.200607020. 17074885 PMC2064519

[67] Topisirovic I., Siddiqui N., Lapointe V.L., Trost M., Thibault P., Bangeranye C., Pinol-Roma S., and Borden K.L. (2009). Molecular dissection of the eukaryotic initiation factor 4E (eIF4E) export-competent RNP. EMBO J 28, 1087–1098. 10.1038/emboj.2009.53. 19262567 PMC2683702

[68] Moissoglu K., Yasuda K., Wang T., Chrisafis G., and Mili S. (2019). Translational regulation of protrusion-localized RNAs involves silencing and clustering after transport. Elife 8. 10.7554/eLife.44752. PMC6639073 31290739

[69] Mardakheh F.K., Paul A., Kumper S., Sadok A., Paterson H., McCarthy A., Yuan Y., and Marshall C.J. (2015). Global Analysis of mRNA, Translation, and Protein Localization: Local Translation Is a Key Regulator of Cell Protrusions. Dev Cell 35, 344–357. 10.1016/j.devcel.2015.10.005. 26555054 PMC4643311

[70] Sanjana N.E., Shalem O., and Zhang F. (2014). Improved vectors and genome-wide libraries for CRISPR screening. Nat Methods 11, 783–784. 10.1038/nmeth.3047. 25075903 PMC4486245

[71] Shalem O., Sanjana N.E., Hartenian E., Shi X., Scott D.A., Mikkelson T., Heckl D., Ebert B.L., Root D.E., Doench J.G., and Zhang F. (2014). Genome-scale CRISPR-Cas9 knockout screening in human cells. Science 343, 84–87. 10.1126/science.1247005. 24336571 PMC4089965

[72] Culjkovic-Kraljacic B., Fernando T.M., Marullo R., Calvo-Vidal N., Verma A., Yang S., Tabbo F., Gaudiano M., Zahreddine H., Goldstein R.L., (2016). Combinatorial targeting of nuclear export and translation of RNA inhibits aggressive B-cell lymphomas. Blood 127, 858–868. 10.1182/blood-2015-05-645069. 26603836 PMC4760090

[73] Zong H., Gozman A., Caldas-Lopes E., Taldone T., Sturgill E., Brennan S., Ochiana S.O., Gomes-DaGama E.M., Sen S., Rodina A., (2015). A Hyperactive Signalosome in Acute Myeloid Leukemia Drives Addiction to a Tumor-Specific Hsp90 Species. Cell Rep 13, 2159–2173. 10.1016/j.celrep.2015.10.073. 26628369 PMC4699804

[74] Sugita M., Galetto R., Zong H., Ewing-Crystal N., Trujillo-Alonso V., Mencia-Trinchant N., Yip W., Filipe S., Lebuhotel C., Gouble A., (2022). Allogeneic TCRalphabeta deficient CAR T-cells targeting CD123 in acute myeloid leukemia. Nat Commun 13, 2227. 10.1038/s41467-022-29668-9. 35484102 PMC9050731

