# Supplementary material for: eIF4E and Ezrin cooperate in pseudopods to drive a localized migratory translation program in acute myeloid leukemia
Source: bioRxiv. 2026 Feb 23:2026.02.21.707190. Preprint. [Version 2] doi: 10.64898/2026.02.21.707190 (PMC13160139; doi:10.64898/2026.02.21.707190)
Supplement: Supplement 1 [file NIHPP2026.02.21.707190v2-supplement-1.pdf]

bioRxiv preprint doi: https://doi.org/10.64898/2026.02.21.707190; this version posted February 23, 2026. The copyright holder for this preprint (which was not certified by peer review) is the author/funder, who has granted bioRxiv a license to display the preprint in perpetuity. It is made available under aCC-BY 4.0 International license.

Supplemental Figure Legends.

bioRxiv preprint doi: https://doi.org/10.64898/2026.02.21.707190; this version posted February 23, 2026. The copyright holder for this preprint (which was not certified by peer review) is the author/funder, who has granted bioRxiv a license to display the preprint in perpetuity. It is made available under aCC-BY 4.0 International license.

Supplemental Figure 1. Characterization of AML cell lines with genetic manipulation of eIF4E. A. Representative western blot from total cell lysates of MM6 and NOMO-1 cell lines demonstrating NOMO-1 cells have similar eIF4E levels to CD34+ from healthy donors, while MM6 cells have similar levels to high-eIF4E AML patient specimens. b-Actin is provided for loading control. Each lane refers to a different sample. **B**. Confocal micrograph of eIF4E nuclear and cytoplasmic staining in MM6 cells. Scale bar = 10 µm. **C, D.** Quantitation of western blots for NOMO-1 eIF4E relative to vector (Figure 1A) or MM6 CRISPR 4E relative to CRISPR-CTRL cells (Figure 1B, Supplemental Figure 1G). Each data point represents a biological replicate. Bar represents the mean. Standard deviation and p-values (Welch's t test) are shown. **E.** Representative confocal micrograph in NOMO-1 eIF4E cells demonstrating that HA staining is specific as its signal is removed upon treatment with hyaluronidase (HAse). HA is red; DAPI is blue. **F.** Representative western blot of MM6 cells treated with the eIF4E inhibitor ribavirin or vehicle control demonstrating that ribavirin reduces eIF4E target proteins including Ezrin. b-Actin is provided as a loading control. **G, H**. Representative western blots demonstrating lower eIF4E levels and factors in the Ezrin-CD44-HA axis in MM6 and THP-1 CRISPR-4E cells compared to CRISPR-Controls.

Supplemental Figure 2. Depletion of eIF4E reduces engraftment and improves overall survival in an MM6 CDX model using an alternative CRISPR/Cas9 clone. A. Schematic of the in vivo assay design. **B.** Validation that depletion of eIF4E decreases AML engraftment in bone marrow and spleen. Gating strategy for the evaluation of human AML cells (top panel). Percent engraftment in bone marrow (left bottom panel) and spleen (right bottom panel). Violin plot representing the median and quartiles, p values calculated with a two tailed Mann-Whitney U test. **C.** Kaplan–Meier curves comparing overall survival of CDX mice with the indicated MM6 cell line clones. Log-rank (Mantel-Cox) test applied. **D.** Western blot analysis of AML cells isolated from

bioRxiv preprint doi: https://doi.org/10.64898/2026.02.21.707190; this version posted February 23, 2026. The copyright holder for this preprint (which was not certified by peer review) is the author/funder, who has granted bioRxiv a license to display the preprint in perpetuity. It is made available under aCC-BY 4.0 International license.

leukemic mice demonstrating that CRISPR 4E MM6 cells have reduced eIF4E levels relative to CRISPR-CTRL, each lane represents a different mouse.

Supplemental Figure 3. Depletion of eIF4E reduces leukemia engraftment in bone marrow and spleen using a THP-1 CDX model. A. Schematic of the in vivo assay design. Engraftment in the bone marrow was assessed on day 30 and 59 post-transplantation. On day 56, a subset of mice (n = 3/cohort) was euthanized to evaluate leukemia engraftment in BM and spleen. Data for day 59 are presented in Figure 2. **B.** Engraftment analysis in BM aspirates form living mice at day 30 post-transplantation. Table shows the number of mice with detectable disease; quantification of THP-1 cells (frequencies) was not possible at this early stage due to their low levels. **C**. Engraftment analysis of a subset of mice at day 56 in bone marrow and spleen. Representative flow cytometry plots show AML cells in bone marrow and spleen from CRISPR 4E and CTRL groups (left panels). Percent of human cells in the indicated organ is shown (right panels), each symbol represents an animal, bars represent the mean, standard deviation and p values (Mann-Whitney U test) shown. **D.** Incidence of abdominal tumor formation at endpoint. Table shows the number of mice developing visible abdominal tumors in each group. **E.** Western blot analysis of AML cells isolated from leukemic mice confirming that THP-1 CRISPR 4E cells maintained reduced eIF4E levels compared with CRISRP-CTRL, each lane represents a different mouse.

Supplemental Figure 4. Pharmacological inhibition of eIF4E impairs invasion and engraftment. A. Invasion capacity of three high-eIF4E AML patient specimens relative to CD34+ cells from healthy donors. Each symbol reflects a different individual. Means and standard deviations, and fold change and p-value (two tailed Welch's t test) per individual AML sample are shown. **B.** Representative dot plots illustrating how the frequency of AML cells was determined using staining with anti-human CD45 (hCD45) and anti-mouse CD45 (mCD45) antibodies. Graphs compare percent of AML cells in peripheral blood in Ribavirin-treated mice (n = 5) and

bioRxiv preprint doi: https://doi.org/10.64898/2026.02.21.707190; this version posted February 23, 2026. The copyright holder for this preprint (which was not certified by peer review) is the author/funder, who has granted bioRxiv a license to display the preprint in perpetuity. It is made available under aCC-BY 4.0 International license.

vehicle-treated controls (n = 5) at 111 and 118 days post-transplantation. Each symbol represents an animal, violin plot representing the median and quartiles, p values calculated with a two tailed Mann-Whitney U test.

Supplemental Figure 5. Cooperation between eIF4E and Ezrin. A. Genetic reduction of Ezrin and eIF4E using siRNA in MM6 cells grown in suspension impacts production of factors in the Ezrin-CD44-HA axis compared to the siLUC control shown in a representative western blot (left panel). b-Actin is provided as loading control. Right panel, quantification of protein expression for the indicated siRNAs relative to siLUC is shown. The expression of each protein was calculated relative to b-Actin. Each symbol represents a biological replicate. Means, standard deviations and p-values (multiple paired t tests). **B.** Endogenous Ezrin and eIF4E immunoprecipitations (IP) in THP-1 total cell lysates show similar observations to MM6 cells (Figure 3C). SN supernatant after immunoprecipitation, IgG negative control. H2B also serves as a negative control for eIF4E and Ezrin IPs. **C.** Cytoplasmic (left) and total (right) cell lysates demonstrate Ezrin immunoprecipitated with the translation machinery including eIF4AI/II. **D.** Example fractionation controls for suspension (left) or invaded (right) cells shown in Figure 3F and G indicating quality of fractions. MEK and CalR are cytoplasmic markers; NOPP140 and H3K27A are nuclear markers.

Supplemental Figure 6. Analysis of T-PODs. A. Schema of VISTA-R for invaded cells. Steps for isolated invaded AML cells for marking active ribosomes with the VISTA-R method. **B.** Confocal microscopy for patient specimen, AML23, demonstrated active translation, characterized by VISTA-R and Ezrin are present in the same pseudopods (white arrow). A single section through the plane of the cell is shown. Scale bar = 10μm. **C.** Representative western blot demonstrating that protein reduction due to siRNA to eIF4E or Ezrin occurs in invaded cells, thus invaded cells are not a result of rescue from the siRNA. Results are similar

bioRxiv preprint doi: https://doi.org/10.64898/2026.02.21.707190; this version posted February 23, 2026. The copyright holder for this preprint (which was not certified by peer review) is the author/funder, who has granted bioRxiv a license to display the preprint in perpetuity. It is made available under aCC-BY 4.0 International license.

to MM6 cells grown in suspension (Figure 3A). **D.** Visualization of masks generated during Imaris confocal analysis to automate identification of pseudopods and subsequent measurement of contents. Scale bar = 10μm. **E.** Total mRNA (left) and rRNA (right) levels detected by RT-qPCR in invaded MM6 cells as a function of genetic knockdown using siRNA to *EIF4E* or *EZR* as compared to siLUC treated cells. Each symbol represents a biological replicate performed independently. Bars represent the mean, shown with standard deviations and p-values (Welch's t test). **F.** Representative western blots of invaded CRISPR MM6 cells showing reduced levels of factors in the Ezrin-CD44-HA axis similar to cells grown in suspension.

bioRxiv preprint doi: https://doi.org/10.64898/2026.02.21.707190; this version posted February 23, 2026. The copyright holder for this preprint (which was not certified by peer review) is the author/funder, who has granted bioRxiv a license to display the preprint in perpetuity. It is made available under aCC-BY 4.0 International license.

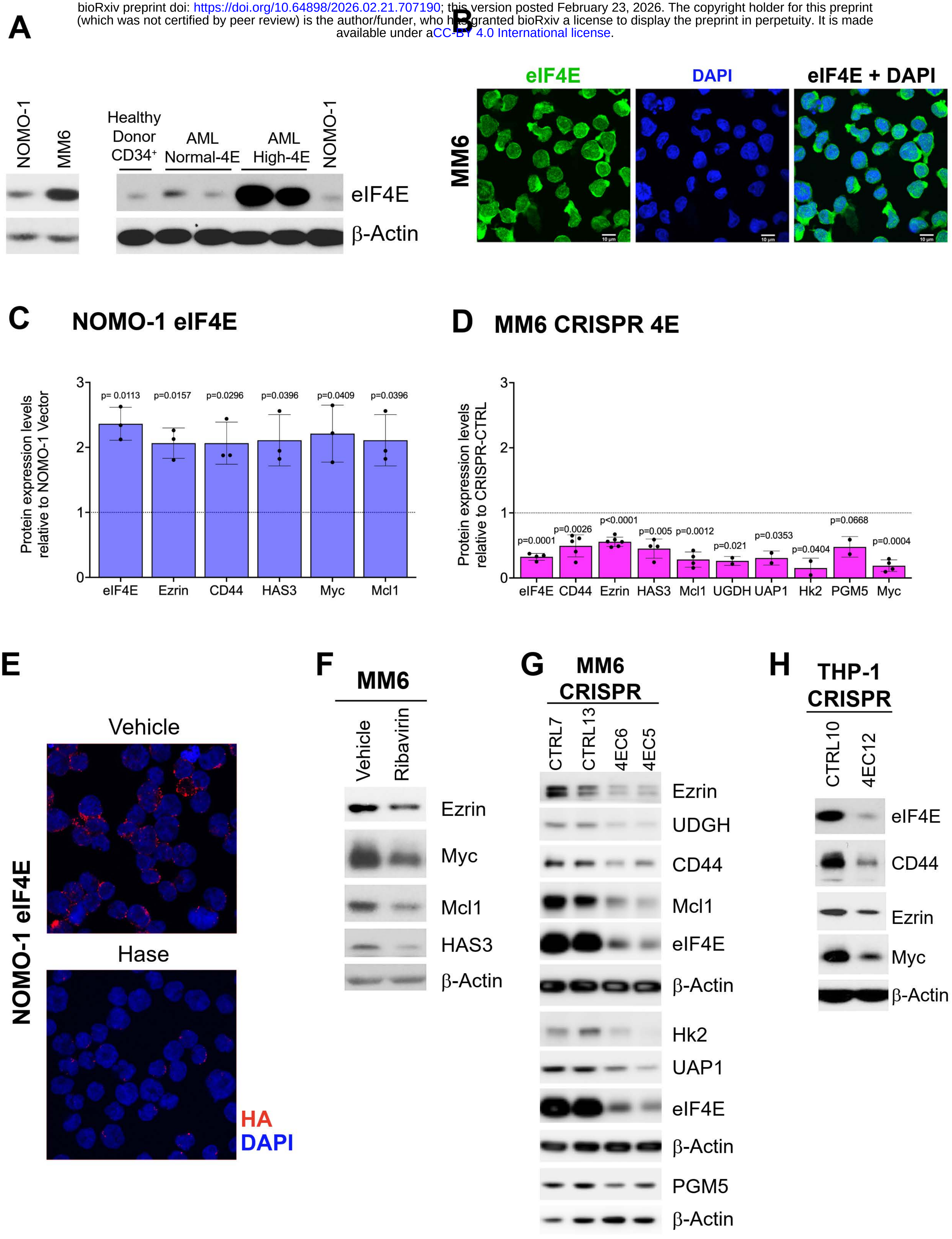

bioRxiv preprint doi: https://doi.org/10.64898/2026.02.21.707190; this version posted February 23, 2026. The copyright holder for this preprint (which was not certified by peer review) is the author/funder, who has granted bioRxiv a license to display the preprint in perpetuity. It is made available under aCC-BY 4.0 International license.

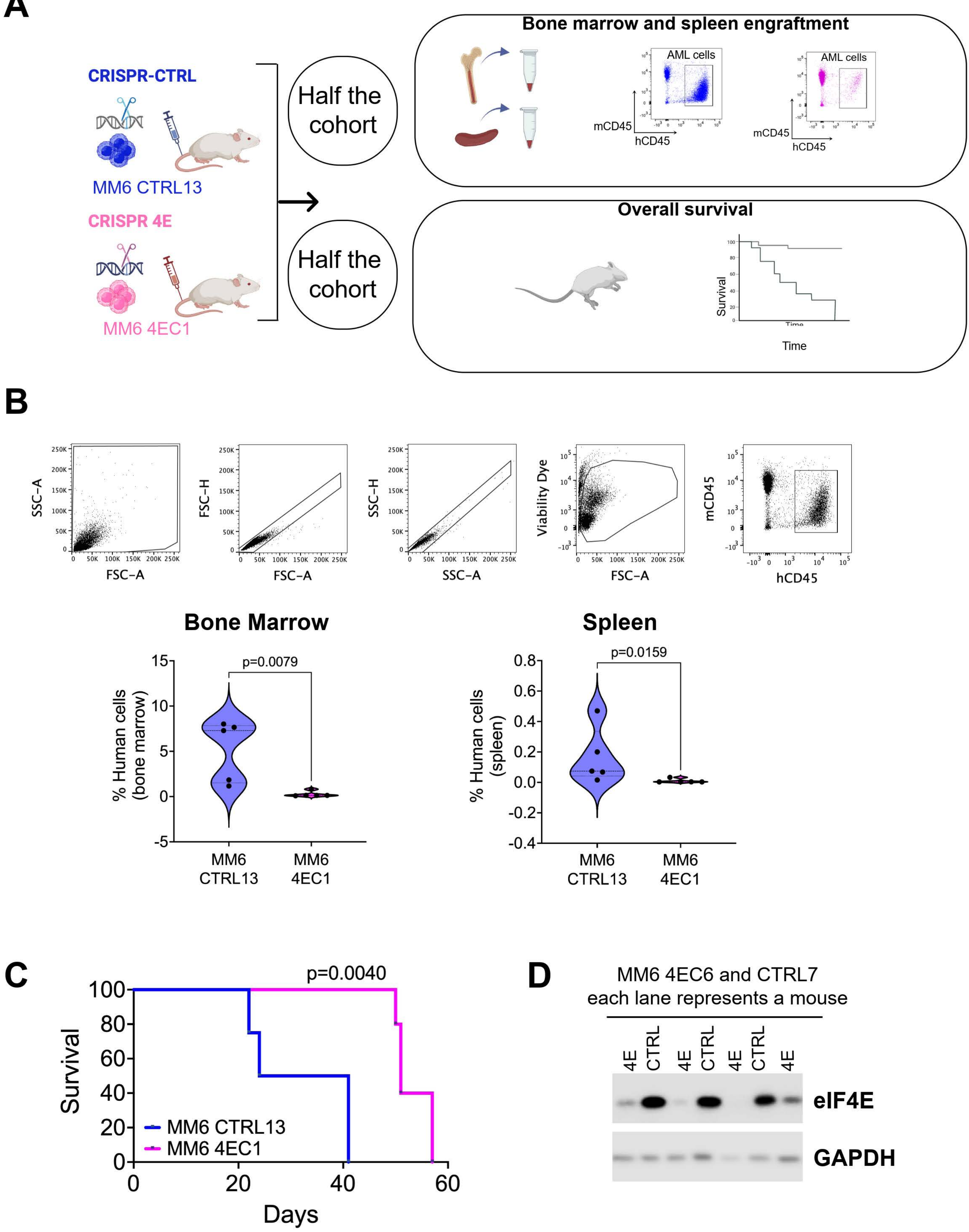

bioRxiv preprint doi: https://doi.org/10.64898/2026.02.21.707190; this version posted February 23, 2026. The copyright holder for this preprint (which was not certified by peer review) is the author/funder, who has granted bioRxiv a license to display the preprint in perpetuity. It is made available under aCC-BY 4.0 International license.

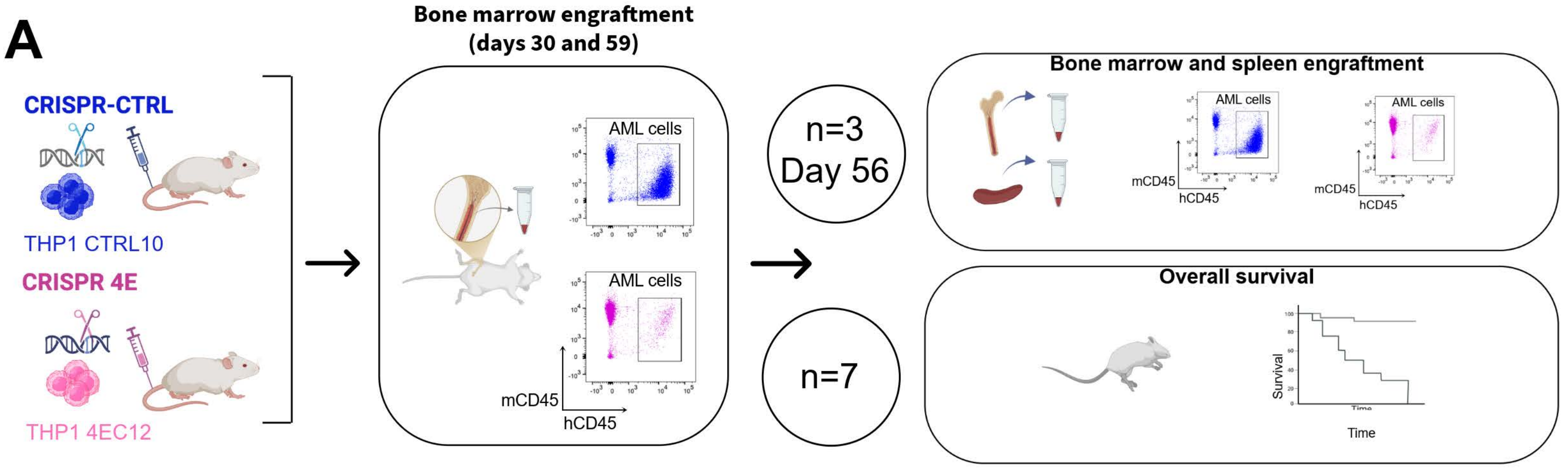

B

	Number of animals with detected human cells	Total
THP-1 CTRL10	8	10
THP-1 4EC12	1	10

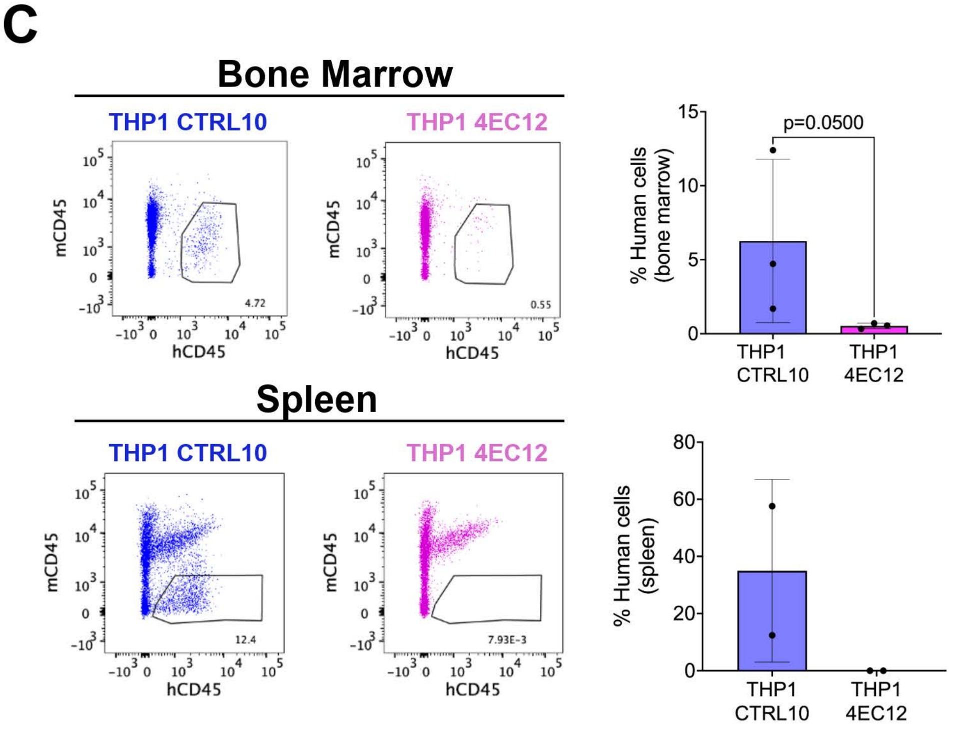

D

	Animals with abdominal tumors at endpoint	Total
THP-1 CTRL10	10	10
THP-1 4EC12	2	10

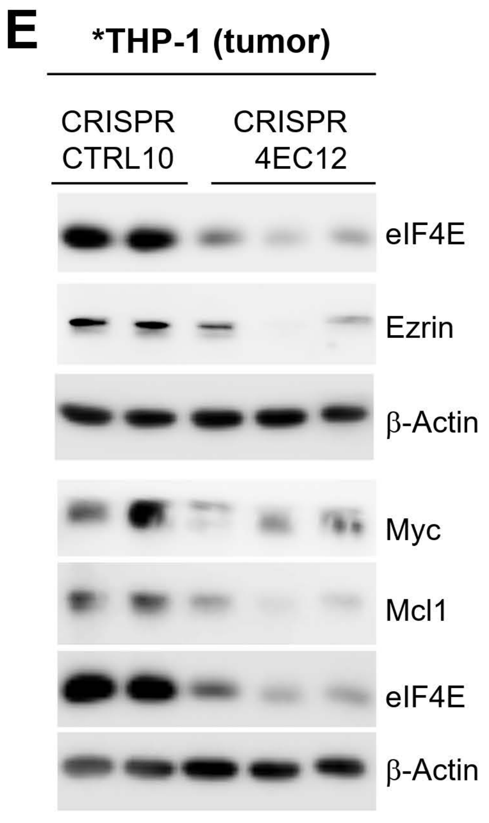

*each lane represents a mouse

bioRxiv preprint doi: https://doi.org/10.64898/2026.02.21.707190; this version posted February 23, 2026. The copyright holder for this preprint (which was not certified by peer review) is the author/funder, who has granted bioRxiv a license to display the preprint in perpetuity. It is made available under a CC-BY 4.0 International license.

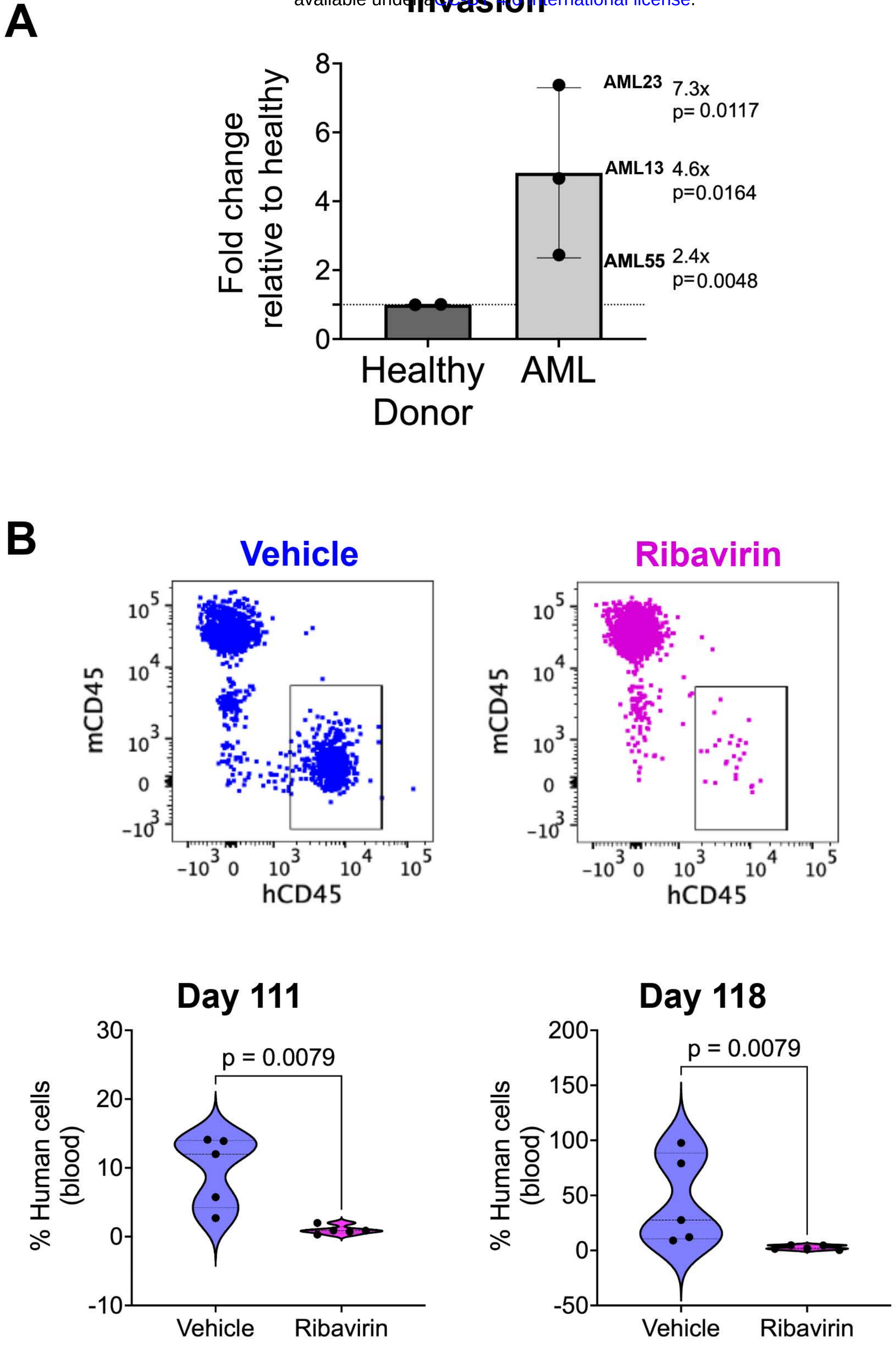

bioRxiv preprint doi: https://doi.org/10.64898/2026.02.21.707190; this version posted February 23, 2026. The copyright holder for this preprint (which was not certified by peer review) is the author/funder, who has granted bioRxiv a license to display the preprint in perpetuity. It is made available under aCC-BY 4.0 International license.

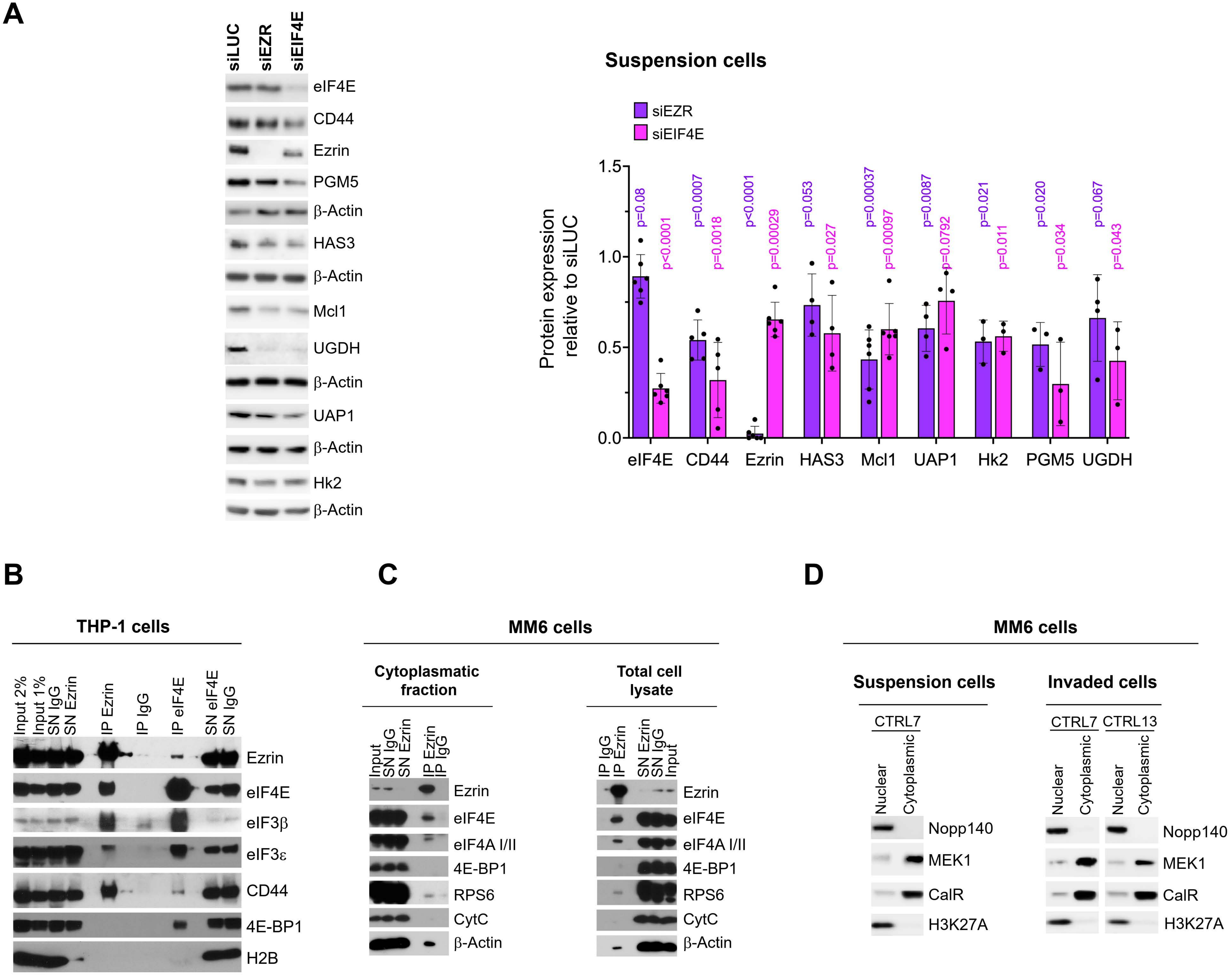

bioRxiv preprint doi: https://doi.org/10.64898/2026.02.21.707190; this version posted February 23, 2026. The copyright holder for this preprint (which was not certified by peer review) is the author/funder, who has granted bioRxiv a license to display the preprint in perpetuity. It is made available under aCC-BY 4.0 International license.

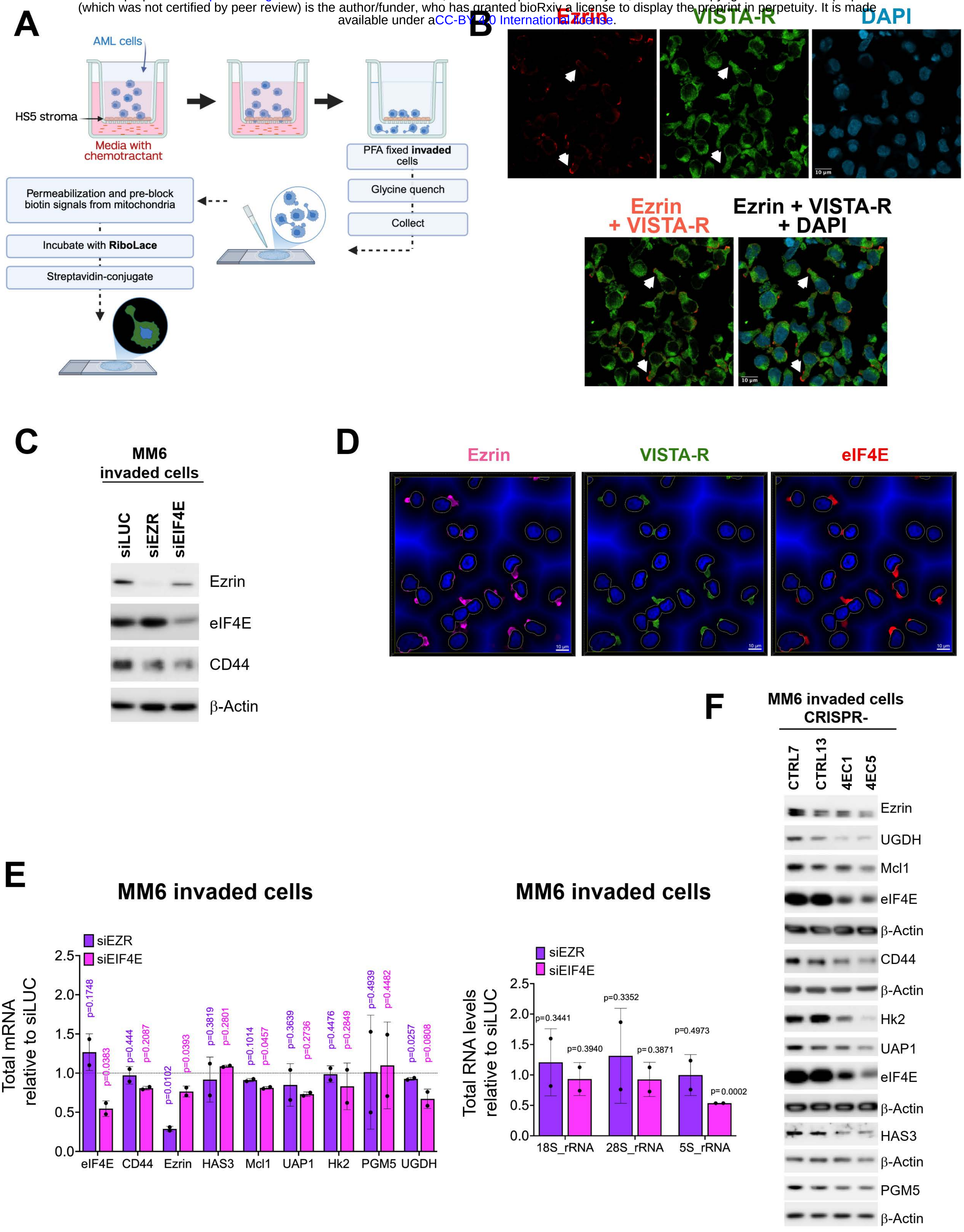

bioRxiv preprint doi: https://doi.org/10.64898/2026.02.21.707190; this version posted February 23, 2026. The copyright holder for this preprint (which was not certified by peer review) is the author/funder, who has granted bioRxiv a license to display the preprint in perpetuity. It is made available under aCC-BY 4.0 International license.

Supplemental Table 1. Primers used in this study.

Name	Sequence
**Mcl1Fw:**	ACTTCTCACTTCCGCTTCCTTCCA
**Mcl1 Rv:**	TTTGAGGCCAAACATTGCCAGTCG
**POL2AFw:**	TGACTGCCAACACAGCCATCTACT
**POL2ARv:**	GGGCCACATCAAAGTCAGGCATTT
**GAPDHFw:**	GAAGGTGAAGGTCGGAGTC
**GAPDHRv:**	GAAGATGGTGATGGGATTTC
**UbcFw:**	ATT TGG GTC GCA GTT CTT G
**UbcRv:**	TGC CTT GAC ATTCTC GAT GGT
**ActinBF:**	GCATGGAGTCCTGTGGCATCCACG
**ActinBR:**	GGTGTAACGCAACTAAGTCATAG
**18S Fw:**	CGG CGA CGA CCC ATT CGA AC
**18S Rv:**	GAA TCG AAC CCT GAT TCC CCG TC
**RPL13aFw:**	TTAATTCCTCATGCGTTGCCTGCC
**RPL13aRv:**	TTCCTTGCTCCCAGCTTCCTATGT
**G6PDHF**	TGGCAAAGTCGGTTTCTCTCTGGA
**G6PDHR**	TTGGGAACATGTCTCAGACTGGCA
**HAS3Fw:**	CAGGAGGACCCTGACTACTT
**HAS3Rv:**	GTGGAAGATGTCCAGCATGTA
**EzrinFw:**	CCGTGGGATGCTCAAAGATAA
**EzrinRv:**	TCCAAGCCAAAGGTCTGTTC
**CD44Fw:**	CGGCTCCTGTTAAATGGTATCT
**CD44Rv:**	TCTGCTTTGTGGTCTGAGAAG
**UAP1Fw:**	GCAGTGCTACAAGGGATCAA
**UAP1Rv:**	CCACCAGCTAGAAGAAGAACTG
**eIF4EFw:**	AGGAGGTTGCTAACCCAGAACACT
**eIF4ERv:**	AAAGTGAGTAGTCACAGCCAGGCA
**HK2Fw:**	TGTGAGGTCCACTCCAGAT
**Hk2Rv:**	GAGCCCATTGTCCGTTACTT
**28SFw:**	GTGCAGATCTTGGTGGTAGTAG
**28SRv:**	TCCAAGCCAAAGGTCTGTTC
**5SFw:**	GGCCATACCACCCTGAAC
**5SRv:**	GGTCTCCCATCCAAGTACTAAC
**PGM5Fw:**	TGATCTCCGAATCGACCTATCT
**PGM5Rv:**	ATATCCACTGGGTCCACTATCT
**mtCox1Fw:**	CAACCTCAACACCACCTTCT
**mtCox1Rv:**	TCCGAAGCCTGGTAGGATAA
**mtCytBFw:**	TCCCCACCCCATCCAACATCT
**mtCytBRv:**	GGTAAAGAATCGTGTGAGGGT
